# ﻿Taxonomic and phylogenetic characterisations of six species of Pleosporales (in Didymosphaeriaceae, Roussoellaceae and Nigrogranaceae) from China

**DOI:** 10.3897/mycokeys.100.109423

**Published:** 2023-11-29

**Authors:** Hongmin Hu, Minghui He, Youpeng Wu, Sihan Long, Xu Zhang, Lili Liu, Xiangchun Shen, Nalin N. Wijayawardene, Zebin Meng, Qingde Long, Jichuan Kang, Qirui Li

**Affiliations:** 1 State Key Laboratory of Functions and Applications of Medicinal Plants, Guizhou Medical University, Guiyang, Guizhou province, China; 2 The High Efficacy Application of Natural Medicinal Resources Engineering Center of Guizhou Province (The Key Laboratory of Optimal Utilization of Natural Medicine Resources), School of Pharmaceutical Sciences, Guizhou Medical University, University Town, Guian New District, Guiyang, Guizhou province, China; 3 Key Laboratory of Infectious Immune and Antibody Engineering of Guizhou Province, Cellular Immunotherapy Engineering Research Center of Guizhou Province, Immune Cells and Antibody Engineering Research Center of Guizhou Province, School of Biology and Engineering, Guizhou Medical University, Guiyang, Guizhou province, China; 4 Center for Yunnan Plateau Biological Resources Protection and Utilization, College of Biological Resource and Food Engineering, Qujing Normal University, Qujing, Yunnan province, China; 5 Tropical Microbiology Research Foundation, 96/N/10, Meemanagoda Road, 10230 Pannipitiya, Sri Lanka; 6 Guizhou Tea Seed Resource Utilization Engineering Research Center, Guizhou Education University, Guiyang, Guizhou province, China; 7 The Engineering and Research Center for Southwest Bio-Pharmaceutical Resources of National Education Ministry of China, Guizhou University, Guiyang, Guizhou province, China

**Keywords:** phylogeny, saprophytic fungi, taxonomy, three new taxa

## Abstract

Pleosporales comprise a diverse group of fungi with a global distribution and significant ecological importance. A survey on Pleosporales (in Didymosphaeriaceae, Roussoellaceae and Nigrogranaceae) in Guizhou Province, China, was conducted. Specimens were identified, based on morphological characteristics and phylogenetic analyses using a dataset composed of ITS, LSU, SSU, *tef*1 and *rpb*2 loci. Maximum Likelihood (ML) and Bayesian analyses were performed. As a result, three new species (*Neokalmusiakarka*, *Nigrogranaschinifolium* and *N.trachycarpus*) have been discovered, along with two new records for China (*Roussoellaneopustulans* and *R.doimaesalongensis*) and a known species (*Roussoellapseudohysterioides*). Morphologically similar species and phylogenetically close taxa are compared and discussed. This study provides detailed information and descriptions of all newly-identified taxa.

## ﻿Introduction

The order Pleosporales was formally established by Luttrell and Barr (1987) and is characterised by perithecioid ascomata with a papillate apex, ostioles with or without periphyses, cellular pseudoparaphyses, bitunicate asci and ascospores of varying shapes, pigmentation and septation (Zhang et al. 2012). As one of the largest orders in the Dothideomycetes, it comprises a quarter of all dothideomycetous species (Ahmed et al. 2014b). Species in this order are found in various habitats and can be epiphytes, endophytes or parasites of living leaves or stems, hyperparasites on fungi or insects, lichenised or saprobes of dead plant stems, leaves or bark (Ramesh 2003; Kruys et al. 2006). In this study, we identified six species belonging to the order Pleosporales from the families Didymosphaeriaceae Munk, Nigrogranaceae Jaklitsch & Voglmayr and Roussoellaceae Jian et al. in Guizhou, China (Wijayawardene et al. 2022).

The family Didymosphaeriaceae, introduced by Munk (1953) and typified by *Didymosphaeriafuckeliana*, can be placed in the order Pleosporales. *Neokalmusia* was introduced to Didymosphaeriaceae by Ariyawansa et al. (2014a). Currently, only eight *Neokalmusia* species are listed in Index Fungorum (accession date: 25 July 2023). Members of Didymosphaeriaceae are known to form numerous different types of life modes, including saprobes, pathogens or endophytes and can be found both on land and in water (Gonçalves et al. 2019; Hongsanan et al. 2020). In the study of this paper, *Neokalmusiakarka* is taken from the dead culms of the *Phragmiteskarka* (Retz.) Trin. ex Steud. Shilihe Beach Park, Huaxi, Guizhou Province, China.

Roussoellaceae was established to accommodate three genera, *Neoroussoella* Jian K. Liu et al., *Roussoella* Sacc. and *Roussoellopsis* I. Hino & Katum., based on molecular phylogenetic studies (Liu et al. 2014). The genus *Roussoella* has cylindrical asci with *Cytoplea* asexual morphs, which distinguishes it from other genera (Liu et al. 2014). Another feature reported for the genus *Roussoella* is the high stability of the ascal exotunica, particularly in 3% potassium hydroxide (KOH). This is quite common for nearly all fungi treated here, while only in *Nigrograna* can fissitunicate ascus dehiscence be seen rather frequently (Jaklitsch and Voglmayr 2016). Nigrogranaceae was established to accommodate *Nigrograna*, with *N.mackinnonii* (Borelli) Gruyter et al. as the type species (Jaklitsch and Voglmayr 2016). As the only genus in the family Nigrogranaceae, *Nigrograna* was established despite lacking strong bootstrap values support in ITS/*tef*1-based phylogenetic trees (Kolařík et al. 2017; Mapook et al. 2020; Zhang et al. 2020a; Wijayawardene et al. 2020). Species of *Nigrograna* may be interpreted as a result of cryptic speciation, as, morphologically, they show only subtle differences (Jaklitsch and Voglmayr 2016). Twenty-three *Nigrograna* species are listed in Index Fungorum (accession date: 25 July 2023).

In this study, we collected dead branches in Guizhou Province, China. Examination of the wood revealed three novel fungal species, two species that are newly recorded in China and one known species of Pleosporales. To elucidate their taxonomic placement and relationships with related species, we conducted morphological observations and phylogenetic analyses, based on combined ITS, LSU, SSU, *tef*1, and *rpb*2 sequences. Detailed descriptions of the morphological features of these species along with their molecular characterisation are provided.

## ﻿Materials and methods

### ﻿Fungal sampling, isolating and morphology

Fresh fungal specimens were collected in Duyun, Zunyi, Qiannan Prefecture and Guiyang, Guizhou Province and were brought back to the laboratory in self-sealing bags. The specimens were then examined for their macroscopic characteristics using a Nikon SMZ 745 series stereomicroscope and photographed, using a Canon 700D digital camera. Micro-morphological structures were photographed using a Nikon digital camera (Canon 700D) that was attached to a light microscope (Nikon Ni). Melzer’s iodine reagent was used to test the apical apparatus structures for amyloid reaction. Measurements of the specimens were registered using Tarosoft (R) Image FrameWork 80 software. The photo plates were arranged and improved using Adobe Photoshop CS6 software. Pure cultures were obtained with the single spore isolation method (Long et al. 2019) and the cultures were grown on potato dextrose agar (PDA) for preservation and observation of the anamorph (Rogers and Ju 1996). The specimens were deposited in the Herbaria of Guizhou Medical University (**GMB**) and Kunming Institute of Botany, Chinese Academy of Sciences (**KUN-HKAS**). Living cultures were deposited at the Guizhou Medical University Culture Collection (**GMBC**).

### ﻿DNA extraction, polymerase chain reaction (PCR) amplification

The pure cultures were cultivated on potato dextrose agar (PDA) medium (Weigh 40.1g of potato dextrose agar (Shanghai Bowei Microbial Technology Co., Ltd.), add 1L of sterile water, and dissolve by heating until boiling. After dissolution, distribute the solution into conical flasks and place them in a high-pressure sterilizer for sterilization. Sterilization conditions are set at 121 degrees Celsius for 30 minutes. After sterilization, add a small amount of injectable potassium penicillin (Huamu) and injectable streptomycin sulfate (Huamu) into the culture medium and mix well. Pour the mixture into disposable culture dishes for later use. This step should be performed in aseptic conditions inside a laminar flow hood.) at 25 °C in the dark for 15–20 days. Fresh mycelium was collected by scraping it with a surgical knife and then transferred to a 1.5 ml centrifuge tube. DNA extraction was performed according to the instructions provided in the Biospin Fungus Genomic DNA Extraction Kit (BIOMIGA®).

The amplification of internal transcribed spacers (ITS), small subunit rDNA (SSU), large subunit rDNA (LSU), translation elongation factor 1-gene region (*tef*1) and RNA polymerase II second largest subunit (*rpb*2) was achieved using ITS5/ITS4, NS1/NS4, LR0R/LR5, EF1-938F/EF1-2218R and fRPB2-5f/fRPB2-7cr primers (Tibpromma et al. 2018; Vu et al. 2019; Wijesinghe et al. 2020; Dissanayake et al. 2021). The polymerase chain reaction (PCR) for the amplification of ITS, SSU, LSU, *tef*1 and *rpb*2 loci were performed using the Eppendorf Mastercycler nexus (SimpliAmp Thermal Cycler, A24811, SimpliAmp, China) gradient under the conditions specified in Table 1. Subsequently, the PCR fragments were sent to Sangon Biotech (Shanghai) Co., China, for sequencing. Amplification conditions using the Polymerase Chain Reaction is shown in Table 2. The obtained sequences were deposited in GenBank and are listed in Table 3.

**Table 1.** PCR conditions used for ITS, SSU, LSU, *tef*1 and *rpb*2 loci.

Genes	Initial period	Cycles, denaturation, annealing and elongation	Final extension
ITS, LSU, SSU, *tef*1	95°C for 5 min	35 cycles of denaturation at 94 °C﻿ for 1 min, annealing at 52﻿°C for 1 min, elongation at 72°C for 1.5 min	72°C for 10 minutes
*rpb*2	95°C for 5 min	35 cycles of denaturation at 95°C for 1 minute, annealing at 54°C for 2 minutes, elongation at 72°C for 1.5 minutes	72°C for 10 minutes

**Table 2.** Composition of PCR reaction system.

Components	Volumetry	Concentration
2× Tap PCR Mix	12.5 μl	1×
Primer 1	1 μl	10μM μl^-1^
Primer	1 μl	10μM μl^-1^
DNA template	1 μl	0.1-0.2 μg μl^-1^
ddH_2_O	Up to 25 μl	

**Table 3.** Taxa and corresponding GenBank accession numbers of sequences used in the phylogenetic analysis of Didymosphaeriaceae, Roussoellaceae and Nigrogranaceae.

Species	Strain	GenBank Accession Numbers					References
		ITS	SSU	LSU	*tef*1	*rpb*2	
*Alloconiothyriumcamelliae*	NTUCC 17-032-1^T^	MT112294	MT071221	MT071270	MT232967	—	(Kolařík et al. 2017)
*Arthopyrenia* sp.	UTHSC DI16–362	LT796905	LN907505	—	LT797145	LT797065	(Crous et al. 2015)
*Austropleosporaochracea*	KUMCC 20-0020^T^	MT799859	MT808321	MT799860	MT872714	—	(Dissanayake et al. 2021)
*A.keteleeriae*	MFLUCC 18-1551^T^	NR_163349	MK347910	NG_070075	MK360045	—	(Mapook et al. 2020)
*Biatriosporaantibiotica*	CCF 1998	LT221894	—	—	—	—	(Kolařík et al. 2017)
*B.carollii*	CCF 4484^T^	LN626657	—	—	LN626668	—	(Kolařík et al. 2017)
*B.mackinnonii*	E9303e	—	—	—	LN626673	—	(Kolařík et al. 2017)
*B.peruviensis*	CCF 4485^T^	LN626658	—	—	LN626671	—	(Kolařík et al. 2017)
*Bimuriaomanensis*	SQUCC 15280^T^	NR_173301	—	NG_071257	MT279046	—	(Wijesinghe et al. 2020)
*B.novae-zelandiae*	CBS 107.79^T^	MH861181	AY016338	AY016356	DQ471087	—	(Vu et al. 2019)
*Chromolaenicolananensis*	MFLUCC 17-1477	MN325014	MN325008	MN325002	MN335647	—	(Liu et al. 2014)
*C.siamensis*	MFLUCC 17-2527^T^	NR_163337	MK347866	NG_066311	MK360048	—	(Mapook et al. 2020)
*C.thailandensis*	MFLUCC 17-1475	MN325019	MN325013	MN325007	MN335652	—	(Liu et al. 2014)
*C.lampangensis*	MFLUCC 17-1462^T^	MN325016	MN325010	MN325004	MN335649	—	(Liu et al. 2014)
*Cylindroaseptosporaleucaenae*	MFLUCC 17-2424	NR_163333	MK347856	NG_066310	MK360047	—	(Mapook et al. 2020)
*Deniquelatahypolithi*	CBS 146988^T^	MZ064429	—	NG_076735	MZ078250	—	(Ariyawansa et al. 2020b)
*D.barringtoniae*	MFLUCC 16-0271	MH275059	—	MH260291	MH412766	—	(Tibpromma et al. 2018)
*Didymocreasadasivanii*	CBS 438.65	MH858658	DQ384066	DQ384103	—	—	(Vu et al. 2019)
*Didymosphaeriarubi-ulmifolii*	MFLUCC 14-0023^T^	—	NG_063557	KJ436586	—	—	(Jayasiri et al. 2019)
*Kalmusiaerioi*	MFLU 18-0832^T^	MN473058	MN473046	MN473052	MN481599	—	(Vu et al. 2019)
*K.italica*	MFLUCC 13-0066^T^	KP325440	KP325442	KP325441	—	—	(Vu et al. 2019)
*K.variisporum*	CBS 121517^T^	NR_145165	—	JX496143	—	—	(Wijesinghe et al. 2020)
*K.ebuli*	CBS 123120^T^	KF796674	JN851818	JN644073	—	—	(Dissanayake et al. 2021)
*Kalmusibambusatriseptata*	MFLUCC 13-0232	KY682697	KY682696	KY682695	—	—	(Tibpromma et al. 2018)
*Karstenularhodostoma*	CBS 690.94	—	GU296154	GU301821	GU349067	—	(Crous et al. 2021)
*Laburnicolahawksworthii*	MFLUCC 13-0602^T^	KU743194	KU743196	KU743195	—	—	(Ariyawansa et al. 2014)
*Letendraeahelminthicola*	CBS 884.85	MK404145	AY016345	AY016362	MK404174	—	(Tibpromma et al. 2018)
*L.muriformis*	MFLUCC 16-0290^T^	KU743197	KU743199	KU743198	KU743213	—	(Ariyawansa et al. 2014)
*L.padouk*	CBS 485.70	—	GU296162	AY849951	—	—	(Zhang et al. 2013)
*L.cordylinicola*	MFLUCC 11 0148^T^	NR_154118	KM214001	NG_059530	—	—	(Wijayawardene et al. 2020)
*Montagnulachromolaenicola*	MFLUCC 17-1469^T^	NR_168866	NG_070157	NG_070948	MT235773	—	(Liu et al. 2014)
*M.cirsii*	MFLUCC 13 0680	KX274242	KX274255	KX274249	KX284707	—	(Hyde et al. 2020)
*M.krabiensis*	MFLUCC 16-0250^T^	MH275070	MH260343	MH260303	MH412776	—	(Tibpromma et al. 2018)
*M.thailandica*	MFLUCC 17-1508^T^	MT214352	NG_070158	NG_070949	MT235774	—	(Liu et al. 2014)
*M.bellevaliae*	MFLUCC 14-0924^T^	NR_155377	KT443904	KT443902	KX949743	—	(Ariyawansa et al. 2014)
*Neoroussoellaalishanense*	FU31016	MK503816	MK503822	—	MK336181	MN037756	(Verkley et al. 2014)
*N.bambusae*	MFLUCC 11–0124	KJ474827	KJ474839	—	KJ474848	KJ474856	(Dissanayake et al. 2021)
*N.brevispora*	KT2313^T^	LC014574	AB524460	AB524601	AB539113	—	(Tanaka et al. 2015)
*N.brevispora*	KT1466	LC014573	AB524459	AB524600	AB539112	—	(Tanaka et al. 2015)
*N.heveae*	MFLUCC 17–1983	MH590693	MH590689	—	—	—	(Wanasinghe et al. 2018)
*N.jonahhulmei*	KUMCC 21-0819	ON007044	ON007040	ON007049	ON009134	—	(Wanasinghe et al. 2016)
***N.karka***	**GMB0494^T^**	**OR120445**	**OR120442**	**OR120432**	**OR150020**	—	**This study**
***N.karka***	**GMB0500**	**OR120438**	**OR120433**	**OR120443**	**OR150021**	—	**This study**
*N.kunmingensis*	KUMCC 18-0120^T^	MK079886	MK079887	MK079889	MK070172	—	(Vu et al. 2019)
*N.lenispora*	GZCC 16-0020^T^	—	KX791431	—	—	—	(Hyde et al. 2020)
*N.scabrispora*	KT1023	LC014575	AB524452	AB524593	AB539106	—	(Tanaka et al. 2015)
*N.solani*	CPC 26331^T^	KX228261	KX228312	—	—	—	(Wijayawardene et al. 2014)
*N.thailandica*	MFLUCC 16-0405^T^	NR_154255	KY706137	NG_059792	KY706145	—	(Thambugala et al. 2015)
*Neokalmusiaarundinis*	MFLUCC 15-0463^T^	NR_165852	NG_068372	NG_068237	KY244024	—	(Thambugala et al. 2015)
*Nigrogranaantibiotica*	CCF 4378^T^	JX570932	—	—	JX570934	—	(Kolařík et al. 2018)
*Nigrogranacangshanensis*	MFLUCC15-0253^T^	KY511063	—	—	KY511066	—	(Crous et al. 2015)
*N.chromolaenae*	MFLUCC 17-1437^T^	MT214379	—	—	MT235801	—	(Liu et al. 2014)
*N.didymospora*	MFLUCC 11-0613	—	KP091435	KP091434	—	—	(Haridas et al. 2020)
*N.fuscidula*	CBS 141556^T^	KX650550	—	—	KX650525	—	(Feng et al. 2019)
*N.fuscidula*	CBS 141476	KX650547	—	—	KX650522	—	(Feng et al. 2019)
*N.hydei*	GZCC 19-0050^T^	NR_172415	—	—	MN389249	—	(Zhang et al. 2020)
*N.impatientis*	GZCC 19-0042^T^	NR_172416	—	—	MN389250	—	(Zhang et al. 2020)
*N.leucaenae*	MFLUCC 18–1544	MK347767	MK347984	—	MK360067	MK434876	(Mapook et al. 2020)
*N.locuta-pollinis*	CGMCC 3.18784	MF939601	—	—	MF939613	—	(Ahmed et al. 2014)
*N.locuta-pollinis*	LC11690	MF939603	—	—	MF939614	—	(Ahmed et al. 2014)
*N.mackinnonii*	CBS 674.75^T^	NR_132037	—	—	KF407986	—	(Ariyawansa et al. 2015)
*N.mackinnonii*	E5202H	JX264157	—	—	JX264154	—	(Phukhamsakda et al. 2018)
*N.magnoliae*	GZCC 17-0057	MF399066	—	—	MF498583	—	(Zhang et al. 2020)
*N.magnoliae*	MFLUCC 20-0020^T^	MT159628	—	—	MT159605	—	(Liu et al. 2014)
*N.mycophila*	CBS 141478^T^	KX650553	—	—	KX650526	—	(Feng et al. 2019)
*N.mycophila*	CBS 141483	KX650555	—	—	KX650528	—	(Feng et al. 2019)
*N.norvegica*	CBS 141485^T^	KX650556	—	—	—	—	(Feng et al. 2019)
*N.obliqua*	CBS 141477^T^	KX650560	—	—	KX650531	—	(Feng et al. 2019)
*N.obliqua*	CBS 141475	KX650558	—	—	KX650530	—	(Feng et al. 2019)
*N.rhizophorae*	MFLUCC 18-0397^T^	MN047085	—	—	MN077064	—	(Poli et al. 2020)
*N.samueliana*	NFCCI-4383^T^	MK358817	—	—	MK330937	—	(Poli et al. 2020)
***N.schinifolium***	**GMB0498^T^**	**OR120434**	—	—	**OR150022**	—	**This study**
***N.schinifolium***	**GMB0504**	**OR120441**	—	—	**OR150023**	—	**This study**
*N.thymi*	MFLUCC 14-1096^T^	KY775576	—	—	KY775578	—	(Crous et al. 2015)
***N.trachycarpus***	**GMB0499^T^**	**OR120437**	—	—	**OR150024**	—	**This study**
***N.trachycarpus***	**GMB0505**	**OR120440**	—	—	**OR150025**	—	**This study**
*N.yasuniana*	YU.101026^T^	HQ108005	—	—	LN626670	—	(Kolařík et al. 2018)
*Occultibambusapustula*	MFLUCC 11-0502^T^	KU940126	—	—	—	—	(Crous et al. 2014)
*O.bambusae*	MFLUCC 13-0855^T^	KU940123	—	—	KU940193	—	(Crous et al. 2014)
*Paracamarosporiumfagi*	CPC 24890^T^	NR_154318	—	NG_070630	—	—	(Ariyawansa et al. 2014)
*P.cyclothyrioides*	CBS 972.95	JX496119	AY642524	JX496232	—	—	(Schoch et al. 2009)
*P.estuarinum*	CBS 109850^T^	JX496016	AY642522	JX496129	—	—	(Verkley et al. 2014)
*P.hawaiiense*	CBS 120025^T^	JX496027	EU295655	JX496140	—	—	(Verkley et al. 2014)
*P.robiniae*	MFLUCC 14–1119^T^	KY511142	KY511141	—	KY549682	—	(Crous et al. 2015)
*P.rosarum*	MFLUCC 17–6054^T^	NR_157529	NG_059872	—	MG829224	—	(Hyde et al. 2016)
*P.rosicola*	MFLUCC 15-0042	NR_157528	MG829153	MG829047	—	—	(Hyde et al. 2016)
*Paramassariosphaeriaanthostomoides*	CBS 615.86	MH862005	GU205246	GU205223	—	—	(Vu et al. 2019)
*Paraphaeosphaeriarosae*	MFLUCC 17-2547^T^	MG828935	MG829150	MG829044	MG829222	—	(Hyde et al. 2016)
*Pararoussoellamukdahanensis*	KUMCC 18-0121	MH453489	MH453485	—	MH453478	MH453482	(Flakus et al. 2019)
*Parathyridariaramulicola*	CBS 141479^T^	KX650565	KX650565	—	KX650536	KX650584	(Feng et al. 2019)
*Phaeodothiswinteri*	CBS 182.58	—	GU296183	GU301857	—	—	(Zhang et al. 2013)
*Pseudocamarosporiumpropinquum*	MFLUCC 13-0544^T^	KJ747049	KJ819949	KJ813280	—	—	(Thambugala et al. 2017)
*Pseudodidymocyrtislobariellae*	KRAM Flakus 25130^T^	NR_169714	NG_070349	NG_068933	—	—	(Tanaka et al. 2015)
*Pseudoneoconiothyriumeuonymi*	CBS 143426^T^	MH107915	MH107961	—	—	MH108007	(Valenzuela-Lopez et al. 2017)
*Pseudopithomycesentadae*	MFLUCC 17-0917^T^	—	MK347835	NG_066305	MK360083	—	(Mapook et al. 2020)
*Pseudoroussoellachromolaenae*	MFLUCC 17–1492^T^	MT214345	MT214439	—	MT235769	—	(Liu et al. 2014)
*P.elaeicola*	MFLUCC 15–0276a	MH742329	MH742326	—	—	—	(Liu et al. 2014)
*P.kunmingnensis*	MFLUCC 17-0314	MF173607	MF173606	MF173605	—	—	(Mapook et al. 2020)
*P.pteleae*	MFLUCC 17-0724^T^	NR_157536	MG829166	MG829061	MG829233	—	(Hyde et al. 2016)
*P.rosae*	MFLUCC 15-0035^T^	MG828953	MG829168	MG829064	—	—	(Hyde et al. 2016)
*P.ulmi-minoris*	MFLUCC 17-0671^T^	NR_157537	MG829167	MG829062	—	—	(Hyde et al. 2016)
*Roussoellaacaciae*	CBS:138873^T^	KP004469	KP004497	—	—	—	(Karunarathna et al. 2019)
*R.aquatic*	MFLUCC 18-1040^T^	NR171975	NG073797	—	—	—	(Liu et al. 2014)
*R.chiangraina*	MFLUCC 10-0556^T^	NR155712	NG059510	—	—	—	(Dissanayake et al. 2021)
*R.doimaesalongensis*	MFLUCC 14-0584^T^	NR165856	NG068241	—	KY651249	KY678394	(Thambugala et al. 2015)
***R.doimaesalongensis***	**GMB0497**	**OR116188**	**OR117732**	—	**OR150026**	—	**This study**
***R.doimaesalongensis***	**GMB0503**	**OR120435**	**OR120444**	—	**OR150027**	—	**This study**
*R.elaeicola*	MFLUCC 15-15-0276a	MH742329	MH742326	—	—	—	(Crous et al. 2015)
*R.euonymi*	CBS:143426^T^	MH107915	MH107961	—	—	MH108007	(Valenzuela-Lopez et al. 2017)
*R.guttulata*	MFLUCC 20-0102^T^	NR172428	NG075383	—	—		(Senwanna et al. 2018)
*R.hysterioides*	CBS 546.94	MH862484	MH874129	—	KF443399	KF443392	(Vilgalys et al. 1990)
*R.intermedia*	CBS 170.96	KF443407	KF443382	—	KF443398	KF443394	(Crous et al. 2013)
*R.japanensis*	MAFF 239636^T^	NR155713	—	—	—	—	(Dissanayake et al. 2021)
*R.kunmingensis*	HKAS 101773^T^	MH453491	MH453487	—	MH453480	MH453484	(Flakus et al. 2019)
*R.magnatum*	MFLUCC 15-0185^T^	—	KT281980	—	—	—	(Jiang et al. 2019)
*R.mangrovei*	MFLU 17-1542^T^	MH025951	MH023318	—	MH028246	MH028250	(Jaklitsch and Voglmayr 2016)
*R.margidorensis*	MUT 5329^T^	NR169906	MN556322	—	MN605897	MN605917	(Tibpromma et al. 2017)
*R.mediterranea*	MUT5369^T^	KU314947	MN556324	—	MN605899	MN605919	(Tibpromma et al. 2017)
*R.mexicana*	CPC 25355^T^	KT950848	KT950862	—	—	—	(Crous et al. 2015a)
*R.mukdahanensis*	MFLU 11-0237^T^	NR155722	—	—	—	—	(Crous et al. 2014)
*R.multiplex*	GMB0316^T^	ON479891	—	ON479892	—	—	(Dong et al. 2020)
*R.neopustulans*	MFLUCC 11-0609^T^	KJ474833	KJ474841	—	KJ474850	—	(Dissanayake et al. 2021)
***R.neopustulans***	**GMB0496**	**OR120436**	**OR120446**				**This study**
***R.neopustulans***	**GMB0502**	**OR116176**	**OR117714**				**This study**
*R.nitidula*	MFLUCC 11-0634	KJ474834	KJ474842	—	KJ474851	KJ474858	(Dissanayake et al. 2021)
*R.padinae*	MUT 5503^T^	—	MN556327	—	MN605902	MN605922	(Tibpromma et al. 2017)
*R.percutanea*	CBS 868.95	KF322118	KF366449	—	KF407987	KF366452	(Ahmed et al. 2014a)
*R.pseudohysterioides*	GMBC0009^T^	MW881445	MW881451	—	—	MW883345	(Zhang et al. 2020)
***R.pseudohysterioides***	**GMB0495**	**OR116175**	**OR117737**	—	**OR150028**	—	**This study**
***R.pseudohysterioides***	**GMB0501**	**OR120447**	**OR120439**	—	**OR150029**	—	**This study**
*R.pustulans*	KT 1709	—	AB524623	—	AB539116	AB539103	(Zhang et al. 2020)
*R.scabrispora*	MFLUCC 14-0582	KY026583	KY000660	—	—	—	(Zhang et al. 2020)
*R.siamensis*	MFLUCC 11-0149^T^	KJ474837	KJ474845	—	KJ474854	KJ474861	(Dissanayake et al. 2021)
*R.thailandica*	MFLUCC 11-0621^T^	KJ474838	KJ474846	—	—	—	(Dissanayake et al. 2021)
*R.tuberculata*	MFLUCC 13-0854^T^	KU940132	KU863121	—	KU940199		(Crous et al. 2014)
*R.verrucispora*	CBS 125434^T^	KJ474832	—	—	—	—	(Dissanayake et al. 2021)
*R.yunnanensis*	HKAS 101762	MH453492	MH453488	—	MH453481	—	(Flakus et al. 2019)
*Roussoellopsismacrospora*	MFLUCC 12-0005	—	KJ474847	—	KJ474855	KJ474862	(Dissanayake et al. 2021)
*R.tosaensis*	KT 1659	—	AB524625	—	AB539117	AB539104	(Zhang et al. 2020)
*Setoarthopyreniachromolaenae*	MFLUCC 17–1444	MT214344	MT214438	—	MT235768	MT235805	(Liu et al. 2014)
*Spegazziniadeightonii*	yone 212	—	AB797292	AB807582	AB808558	—	(Tanaka et al. 2015)
*S.radermacherae*	MFLUCC 17-2285^T^	MK347740	MK347848	MK347957	MK360088	—	(Mapook et al. 2020)
*S.tessarthra*	NRRL 54913	JQ673429	AB797294	AB807584	AB808560	—	(Tanaka et al. 2015)
*Thyridariaacaciae*	CBS 138873	KP004469	KP004497	—	—	—	(Liu et al. 2014)
*T.broussonetiae*	CBS 141481	NR_147658	KX650568	—	KX650539	KX650586	(Karunarathna et al. 2019)
*Torulaherbarum*	CBS 111855	KF443409	KF443386	—	KF443403	KF443396	(Crous et al. 2013)
*T.hollandica*	CBS 220.69	KF443406	KF443384	—	—	KF443393	(Crous et al. 2013)
*Tremateiaarundicola*	MFLU 16-1275	KX274241	KX274254	KX274248	KX284706	—	(Hyde et al. 2020)
*T.chromolaenae*	MFLUCC 17-1425^T^	NR_168868	NG_070160	NG_068710	MT235778	—	(Tanaka et al. 2015)
*T.guiyangensis*	GZAAS01	KX274240	KX274253	KX274247	KX284705	—	(Hyde et al. 2020)
*T.murispora*	GZCC 18-2787	NR_165916	MK972750	MK972751	MK986482	—	(Feng et al. 2019)
*T.thailandensis*	MFLUCC 17-1430^T^	NR_168869	NG_070161	NG_068711	MT235781	—	(Liu et al. 2014)
*Verrucoconiothyriumnitidae*	CBS:119209	EU552112	—	EU552112	—	—	(Wanasinghe et al. 2018)
*Xenocamarosporiumacaciae*	CPC 24755^T^	NR_137982	—	NG_058163	—	—	(Crous et al. 2015b)
*Xenoroussoellatriseptata*	MFLUCC 17–1438	MT214343	MT214437	—	MT235767	MT235804	(Liu et al. 2014)

**Notes**: Type specimens or Ex-type specimens are marked with T; “–”: indicates no sequence available in GenBank; newly-generated sequences are indicated in bold. **Abbreviations: CBS**: Centraalbureau voor Schimmelcultures, Utrecht, The Netherlands; **CPC**: Culture collection of Pedro Crous, housed at the Westerdijk Fungal Biodiversity Institute; **GMB**: Culture collection of Guizhou Medical University; **HKAS**: Herbarium of Cryptogams Kunming Institute of Botany Academia Sinica, Chinese Academy of Sciences, Kunming, China; **HKUCC**: Hong Kong University Culture Collection; **KT**: K. Tanaka; **KUMCC**: Kunming Institute of Botany Culture Collection, Chinese Science Academy, Kunming, China; **MAFF**: Ministry of Agriculture, Forestry and Fisheries, Japan; **MFLUCC**: Mae Fah Luang University Culture Collection, Chiang Rai, Thailand; **NFCCI**: National Fungal Culture Collection of India; **Others**: information not available.

### ﻿Phylogenetic analysis

BioEdit v.7.0 was used to verify the quality of sequences (Hall TA 1999) and MAFFT v.7.215 (http://mafft.cbrc.jp/alignment/server/index.html) was employed to generate single gene alignments (Katoh and Standley 2013). The file format was converted using ALTER (Alignment Transformation Environment) (http://www.sing-group.org/ALTER/). Maximum Likelihood (ML) analyses and Bayesian posterior probabilities (BYPP), based on a combination of ITS, LSU, *tef*1 and *rpb*2 sequence data, were performed using RAxML-HPC BlackBox and MrBayes v. 3.2.7a tools in the CIPRES Science Gateway platform (Liang et al. 2020). GTR+I+G was estimated as the best-fit substitution model by ModelTest2 on XSEDE v.2.1.6. (Posada and Crandall 1998).

Bayesian Inference (BI) analysis was conducted using MrBayes v.3.2.7a (Ronquist et al. 2012) and posterior probabilities (PP) were determined through Markov Chain Monte Carlo sampling (MCMC). Six simultaneous Markov chains for 3,000,000 generations were run and trees were sampled every 1,000^th^ generation.

The trees were visualised using FigTree v,1.4.4, and formatted using Adobe Illustrator CS v.6. Branches with Maximum-Likelihood bootstrap values (MLBP) equal to or greater than 75% and Bayesian posterior probabilities (BYPP) greater than 0.95 are indicated. The combined loci alignment and resulting phylogenetic trees were submitted to TreeBASE (https://www.treebase.org, submission number: ID 30482; ID 30483; ID 30484).

## ﻿Results

### ﻿Phylogenetic analyses

**Figure 1.**
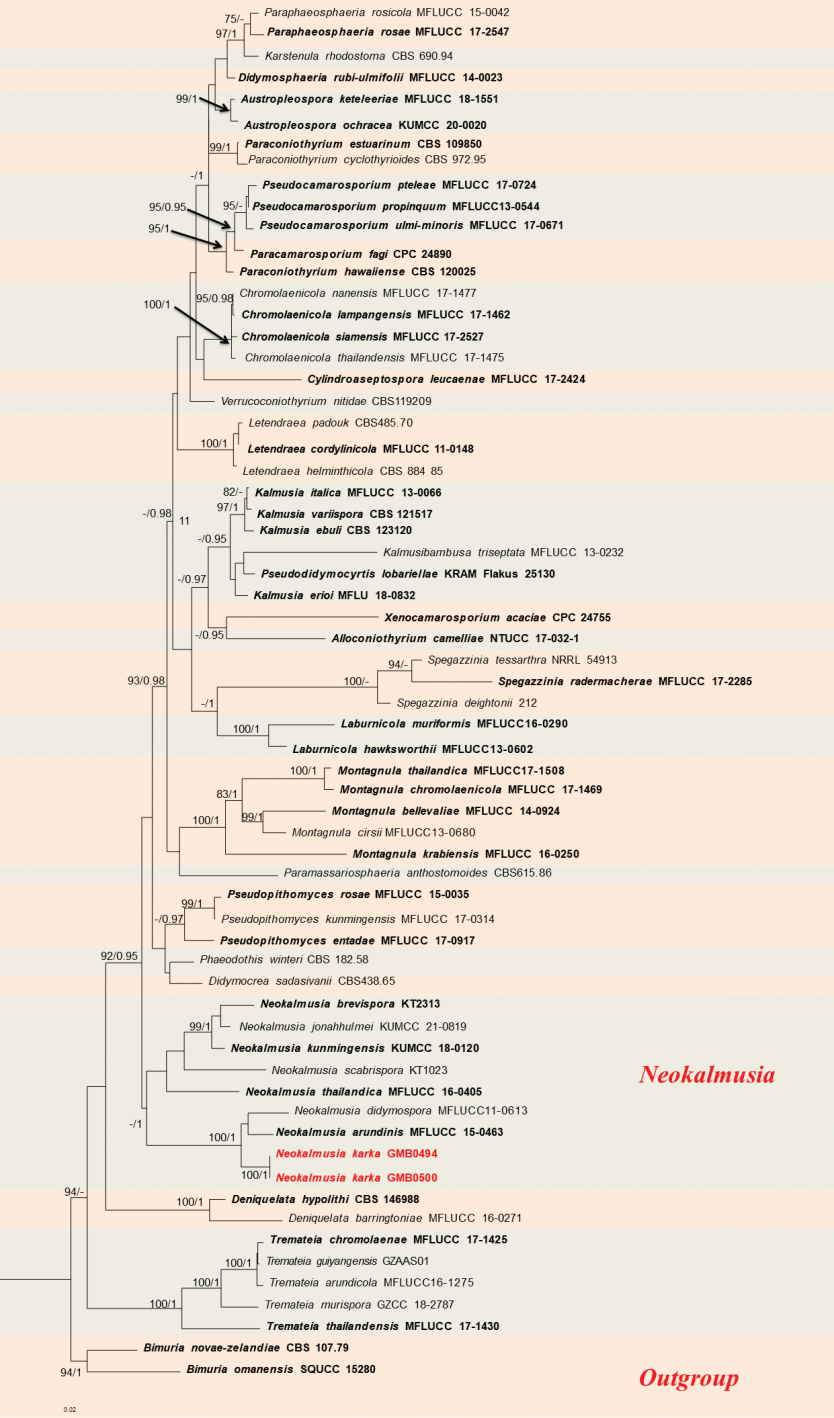
RAxML phylogram of Didymosphaeriaceae, based on a combined dataset of partial ITS, LSU, SSU and *tef*1 DNA sequences. The tree is rooted by *Bimurianovae-zelandiae* (CBS 107.79) and *Bimuriaomanensis* (SQUCC 15280). Bootstrap supports ML (MLB ≥ 75%) and Bayesian posterior probabilities (BYPP ≥ 0.95) are given as MLB/BYPP above the branches. Sequences from newly-generated isolates are in red, bold letters, while those of ex-type isolates are shown in black, bold letters.

**Figure 2.**
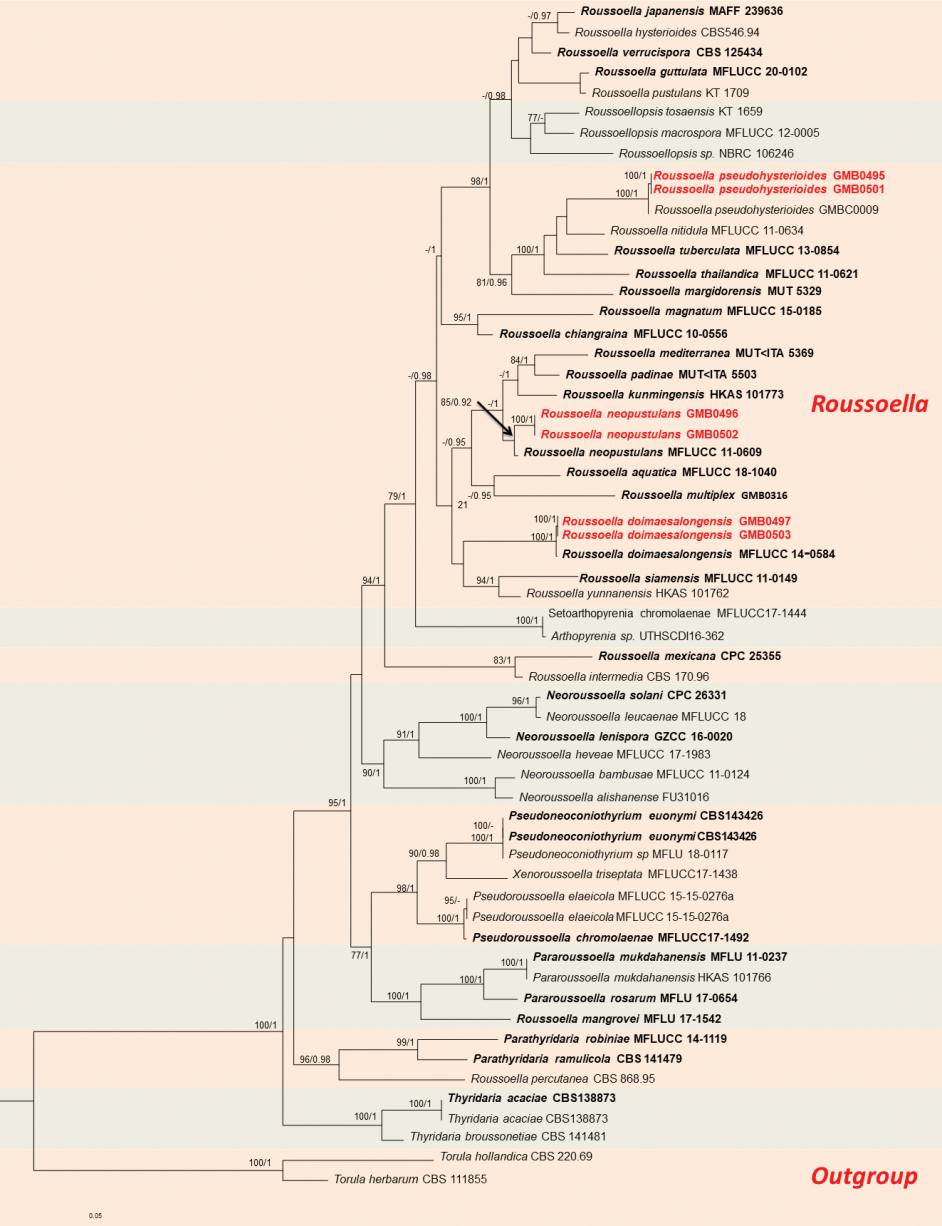
RAxML phylogram of Roussoellaceae, based on a combined dataset of partial ITS, LSU, *tef*1 and *rpb*2 DNA sequences. The tree is rooted by *Torulahollandica* (CBS 220.69) and *T.herbarum* (CBS 111855). Bootstrap supports ML (MLB ≥ 75%) and Bayesian posterior probabilities (BYPP ≥ 0.95) are given as MLB/BYPP above the branches. Sequences from newly-generated isolates are in red, bold letters, while those of ex-type isolates are shown in black, bold letters.

**Figure 3.**
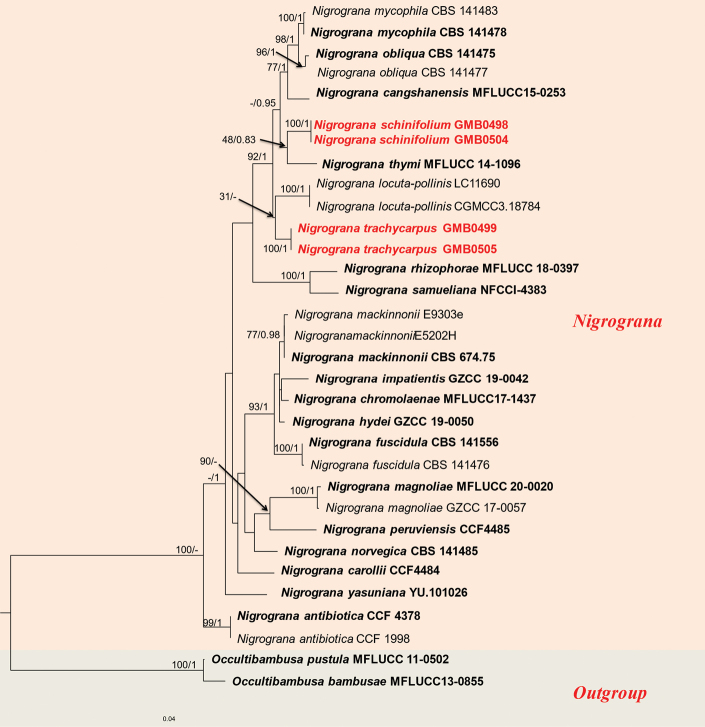
RAxML phylogram of Nigrogranaceae, based on a combined dataset of ITS and *tef*1 DNA sequences. The tree is rooted by *Occultibambusapustula* (MFLUCC 11-0502) and *O.bambusae* (MFLUCC 13-0855). Bootstrap supports ML (MLB ≥ 75%) and Bayesian posterior probabilities (BYPP ≥ 0.95) are given as MLB/BYPP above the branches. Sequences from newly-generated isolates are in red, bold letters, while those of ex-type isolates are shown in black, bold letters.

Phylogenetic analyses of Didymosphaeriaceae (Fig. 1), Roussoellaceae (Fig. 2), and Nigrogranaceae (Fig. 3) were performed separately, with corresponding parameters presented in Table 4.

**Table 4.** Results of Maximum-Likelihood (ML) and Bayesian (BI) analyses for each sequenced dataset.

Analyses		Didymosphaeriaceae	Roussoellaceae	Nigrogranaceae
Number of taxa		64	59	32
Gene regions		ITS, LSU, SSU and *tef*1	ITS, LSU, *tef*1 and *rpb*2	ITS and *tef*1
Number of character positions (including gaps)		2423	2267	868
ML optimisation likelihood value		-13324.603084	-16237.062124	-3695.409391
Distinct alignment patterns in the matrix		584	773	240
Number of undetermined characters or gaps (%)		14.26%	27.45%	7.87%
Estimated base frequencies	A	0.237970	0.240773	0.229686
	C	0.246811	0.255815	0.293625
	G	0.277468	0.276383	0.242370
	T	0.237752	0.227030	0.234319
Substitution rates	AC	1.764988	2.186105	1.598706
	AG	2.187844	5.410475	2.533043
	AT	1.416956	2.441301	1.640025
	CG	1.132266	1.384067	0.752494
	CT	7.848138	11.885781	8.062830
	GT	1.000000	1.000000	1.000000
Proportion of invariable sites (I)		0.595845	0.544120	0.487317
Gamma distribution shape parameter (a)		0.516792	0.502253	0.634309
Number of generated trees in BI		14806	10678	9932
Average standard deviation of split frequencies		0.006852	0.004431	0.004939

### ﻿Taxonomy

**Didymosphaeriaceae Munk, 1953**

#### ﻿ Neokalmusia

Taxon classificationFungiPleosporalesDidymosphaeriaceae

Ariyawansa & K.D. Hyde, Fungal Diversity 68: 92 (2014b)

99794A31-D28F-589E-BC9A-E7EA035988C5

550700

##### Notes.

*Neokalmusia* was established by Ariyawansa et al. (2014b) to accommodate two bambusicolous taxa, *N.brevispora* and *N.scabrispora*, previously referred to *Kalmusia*. Members of *Neokalmusia* are characterised by solitary sphaeroid ascomata, a peridium of small pseudoparenchymatous cells, clavate basal asci with very long pedicels, very thin pseudoparaphyses and distoseptate, smooth-walled ascospores (Ariyawansa et al. 2014b; Zhang et al. 2020a). In this study, we introduce a new species of *Neokalmusia*, based on a combination of morphological and molecular analyses (Fig. 1).

#### ﻿ Neokalmusia karka

Taxon classificationFungiPleosporalesDidymosphaeriaceae

H. M. Hu & Q. R. Li sp. nov.

08F65237-C479-55E6-9281-AA50B473E330

851046

Fig. 4

##### Type material.

***Holotype***: GMB0494.

##### Etymology.

In reference to the host, *Phragmiteskarka* (Retz.) Trin. ex Steud.

##### Description.

Saprobic on dead culms of *P.karka*.

**Sexual morph: *Clypeus*** visible as black dots on the host surface, breaking through slightly raised cracks at the centre. ***Ascomata*** 241–386 × 161–231 μm (average = 375 × 197 μm, n = 5), smooth, semi-immersed, scattered, solitary or in small groups, black, oval, with ostiole. ***Peridium*** 12–20 μm wide, composed of a few layers of thin-walled, brown to dark brown, cells of textura angularis. ***Hamathecium*** comprising 1.5–2.8 μm wide, numerous, cellular, pseudoparaphyses, embedded in a mucilaginous matrix. ***Asci*** 80–109 × 10–14 μm (average = 95 × 11.4 μm, n = 15), 8-spored, bitunicate, fissitunicate, cylindrical-clavate, with bulbous pedicel, apically rounded with an indistinct ocular chamber, with a J-subapical ring. ***Ascospores*** 14–17 × 4–6 μm (average = 15.8 × 5.3 μm, n = 30), overlapping 1–2-seriate, fusiform, pale brown to brown, 1-septate, constricted at the septum, often enlarged near septum in the upper cell, distinctly verrucose on the surface, without a mucilaginous sheath. **Asexual morph**: undetermined.

##### Culture characters.

After 4 weeks of cultivation at 25 °C, the colonies on PDA measure around 2–2.5 cm in diameter. The surface appears smooth to velvety with an entire or slightly irregular margin, ranging from white to grey olivaceous. The colour is white near the margin with dense circular to filamentous growth. The reverse side of the colonies black to greenish-olivaceous.

##### Specimens examined.

China, Guizhou Province, Zunyi City, Suiyang County, Kuanqwashui Nature Reserve (28°31'51.04"N, 107°9'33.65"E), 1544 m elev., on decaying culms, 12 October 2022, Y.P Wu and H.M Hu, 2022KKS49 (GMB0494, holotype; GMBC0494, ex-type; KUN-HKAS 129179, isotype).

##### Other examined material.

China, Guizhou Province, Huaxi District, Shilihetan Wetland Park (26°41'34.3"N, 106°67'68.8"E), 1500 m elev., on decaying culms, 8 October 2022, Y.P Wu and H.M Hu, 2022SLZH11 (GMB0500; GMBC0500, living culture).

##### Notes.

This fungus shares morphological characters similar to *Neokalmusia* in having immersed ascomata, a clypeus-like structure composed of thin-walled cells and verrucose ascospores (Tanaka et al. 2009; Ariyawansa et al. 2014b). Other than *Neokalmusiakarka*, only two species, *N.arundinis* Thambug. & K.D. Hyde and *N.didymospora* D.Q. Dai & K.D. Hyde have been reported with 1-septate ascospores. However, *N.karka* can be distinguished, based on differences in asci size (*N.karka*, 80–109 × 10–14 μm; *N.arundinis* 60–85 × (7.5–) 8.5–10.5 μm; *N.didymospora* 125–160 × 9.5–14 μm) and the obvious oval shape of its ascomata (Wanasinghe et al. 2018; Flakus et al. 2019). In our phylogram, *Neokalmusiakarka* formed a well-supported separate clade (100% ML, 1 BYPP; Fig. 1) in a sister relationship with *N.arundinis* and *N.didymospora*. The macro and micro-morphological differences and phylogenetic analyses support the recognition of *N.karka* as a new species (Fig. 1).

#### Roussoellaceae Jian K. Liu, Phook., D.Q. Dai & K.D. Hyde 2014

##### ﻿ Roussoella

Taxon classificationFungiPleosporalesRoussoellaceae

Sacc., Atti Inst. Veneto Sci. lett., ed Arti, Sér. 6 6: 410 (1888)

87332ACB-77A1-5002-ABE6-CEA873335CE1

541317

###### Notes.

the genus *Roussoella* was introduced by Saccardo et al. (1888), with *R.nitidula* Sacc. & Paol. as the type species, which was collected from bamboo in Malaysia. This family is characterised as having semi-immersed to immersed, solitary or gregarious, clypeate ascostromata containing trabeculate pseudoparaphyses embedded in a gel matrix, long cylindrical to clavate bitunicate asci with or without obvious fissitunicate dehiscence and brown, 2-celled ornamented ascospores (Liu et al. 2014). In this study, we introduce three new records of *Roussoella* species, based on morpho-anatomical and molecular analyses (Fig. 2).

**Figure 4.**
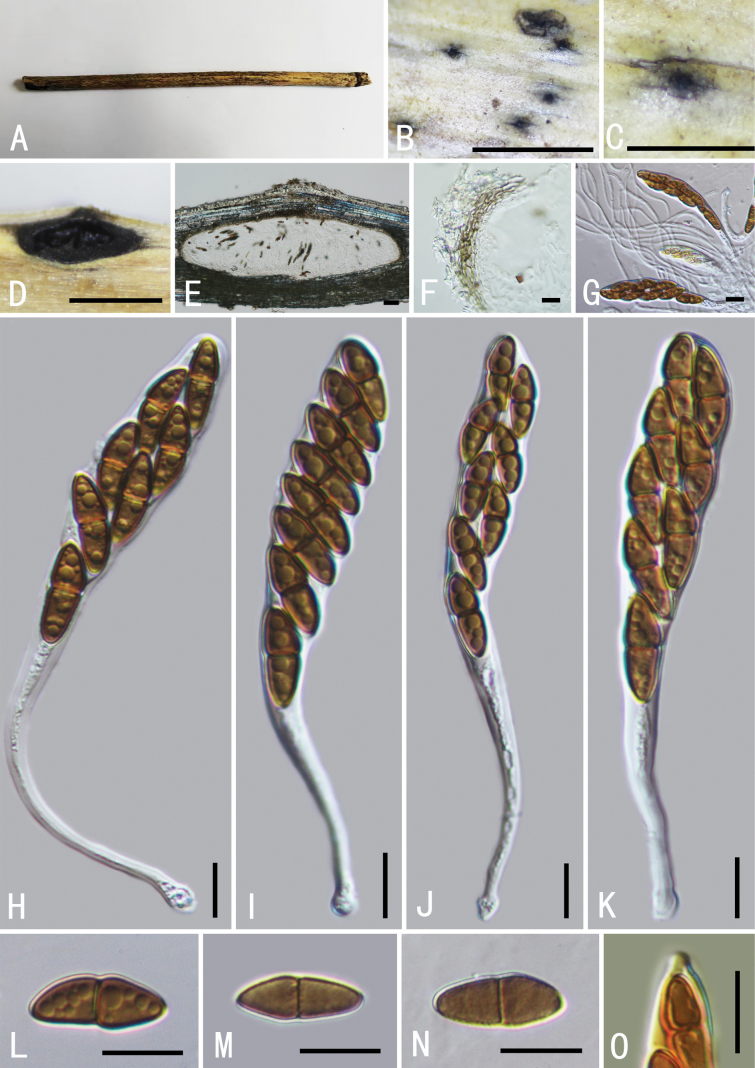
*Neokalmusiakarka* (GMB0494, holotype) **A** type specimen **B**, **C** appearance of ascomata on substrate **D, E** longitudinal section of an ascoma **F** peridium **G** pseudoparaphyses **H–K** asci **L–N** ascospores **O, J** ascus subapical ring in Melzer’s Reagent. Scale bars: 0.5 mm (**B–D**); 10 μm (**E–O**).

##### ﻿ Roussoella pseudohysterioides

Taxon classificationFungiPleosporalesRoussoellaceae

D.Q. Dai & K.D. Hyde, in Dai et al., Fungal Diversity 82(1): 37 (2017)

C1CF6CE5-DC0E-531C-9BCC-8E0B06A93B1C

552026

Fig. 5

###### Descriptions.

See Dai et al. (2017).

###### Specimen examined.

China, Guizhou Province, Huaxi District, Shilihetan Wetland Park (26°43'34.3"N, 106°67'68.8"E), 1542 m elev., on decaying bamboo, 8 October 2022, Y.P Wu and H.M Hu, 2022SLZH6 (GMB0495; GMBC0495, living culture).

**Figure 5.**
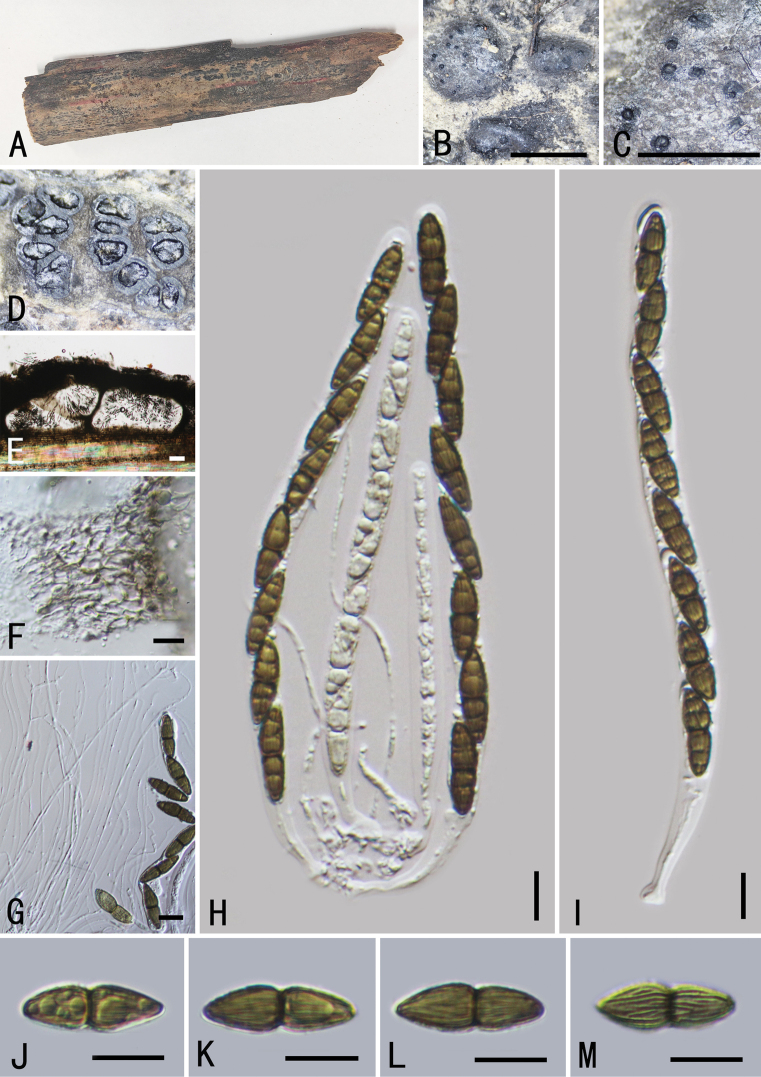
*Roussoellapseudohysterioides* (GMB0495) **A** specimen **B, C** appearance of ascomata on substrate **D** cross-section of ascostromata **E** longitudinal section of an ascoma **F** peridium **G** pseudoparaphyses **H–I** asci **J–M** ascospores. Scale bars: 0.5 mm (**B–D)**; 10 μm (**E–M**).

###### Notes.

Phylogenetic analyses of the combined ITS, LSU, *tef*1 and *rpb*2 gene sequences showed that the sequence from our 2022SLZH6 collection clusters together with *Roussoellapseudohysterioides* (MFLU 15-1209), with strong support (100% ML, 1 BYPP; Fig. 2). The morphological characteristics of our specimen are also consistent with those of *R.pseudohysterioides*, which was originally described from decaying bamboo culms in Thailand (Dai et al. 2017). In China, it had previously been reported from Yunnan Province (Jiang et al. 2019). This is the second report of this species in China, representing a new record for Guizhou Province.

##### ﻿ Roussoella neopustulans

Taxon classificationFungiPleosporalesRoussoellaceae

D.Q. Dai, J.K. Liu & K.D. Hyde, in Liu et al. Phytotaxa 181(1): 15 (2014)

5A992DD1-B7DD-50AC-AD02-0852427EDF6C

550664

Fig. 6

###### Descriptions.

See Liu et al. (2014).

###### Specimens examined.

China, Guizhou Province, Huaxi District, Guiyang Huaxi National Urban Wetland Park (26°2'2.34"N, 106°34'16.22"E), on dead branch of bamboo, 12 October 2022, 1130 m elev., Y.P Wu and H.M Hu, 2022HX25 (GMB0496; GMBC0496, living culture).

**Figure 6.**
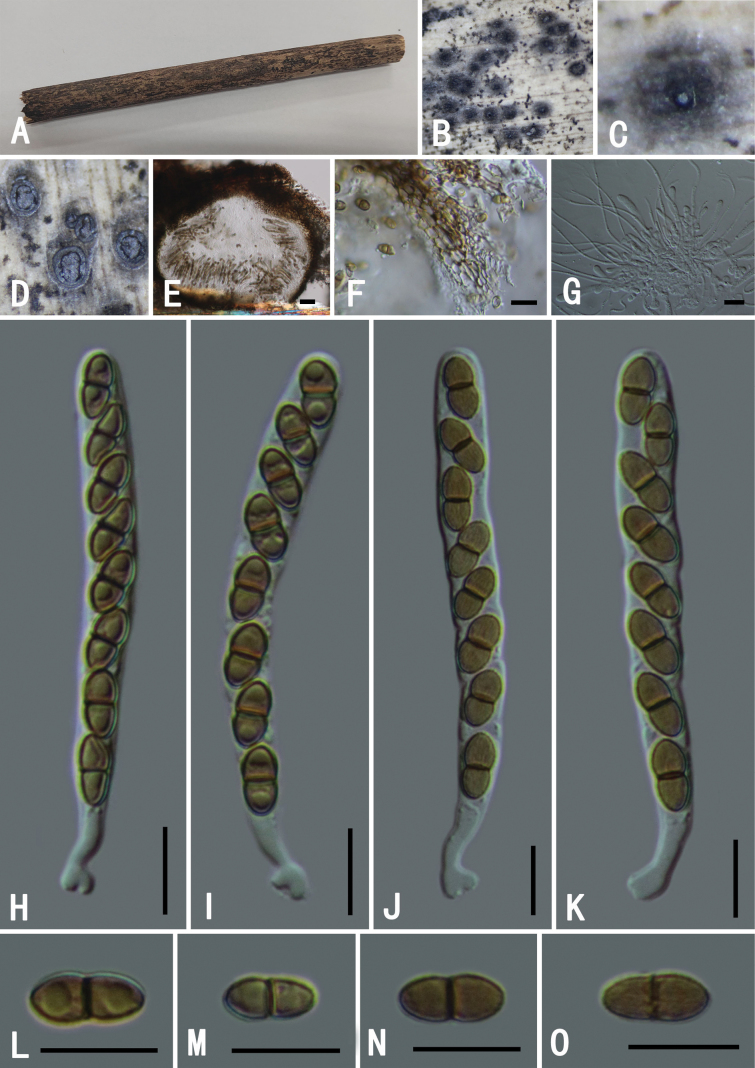
*Roussoellaneopustulans* (GMB0496) **A** specimen **B, C** appearance of ascomata on substrate **D** cross-section of ascostromata **E** longitudinal section of an ascoma **F** peridium **G** pseudoparaphyses **H–K** asci **L–O** ascospores. Scale bars: 0.5 mm (**B–D**); 10 μm (**E–O**).

###### Notes.

The sequence of our *Roussoellaneopustulans* (2022HX25) forms a well-supported clade (85% ML, 0.92 BYPP; Fig. 2) with *R.neopustulan* (MFLUCC 11-0609). *Roussoellaneopustulans* was originally introduced by Liu et al. (2014), with a description of the sexual morph only. Dai et al. (2017) provided a comprehensive description and illustrations for both the sexual and asexual morphs of this species. Our collection exhibits identical morphological characteristics to those detailed by Dai et al. (2017). This is the first report of this species in China.

##### ﻿ Roussoella doimaesalongensis

Taxon classificationFungiPleosporalesRoussoellaceae

Thambug. & K.D. Hyde, Mycosphere 8 (4): 782 (2017)

96CCCA92-3399-57FD-80F8-D8B4D725B0CF

553169

Fig. 7

###### Descriptions.

See Thambugala et al. (2017).

###### Specimen examined.

China, Guizhou Province, Huaxi District, Shilihetan Wetland Park (26°23'23.4"N, 106°67'56.4"E), 1511 m elev., on dead bamboo branches, 8 October 2022, Y.P Wu and H.M Hu, 2022SLHT14 (GMB0497; GMBC0497, living culture).

**Figure 7.**
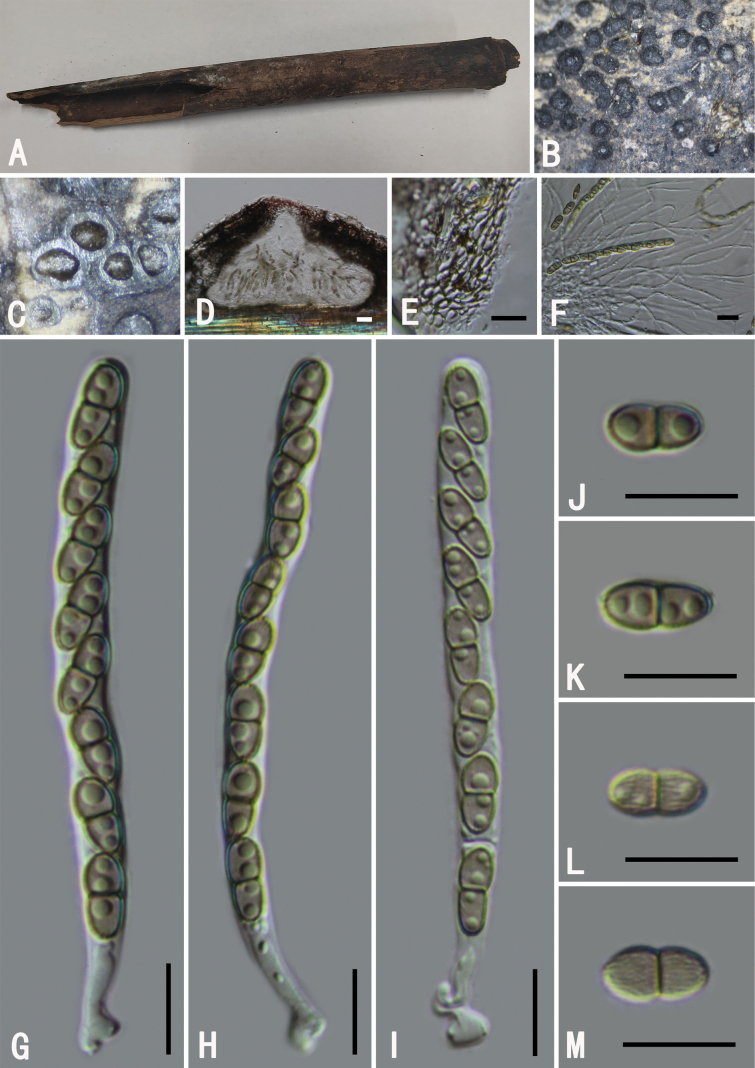
*Roussoelladoimaesalongensis* (GMB0497) **A** specimen **B** appearance of ascomata on substrate **C** cross-section of ascostromata **D** longitudinal section of an ascoma **E** peridium **F** pseudoparaphyses **G–I** asci **J–M** ascospores. Scale bars: 0.5 mm (**B–C)**; 10 μm (**D–M)**.

###### Notes.

In our phylogram (Fig. 2), the sequence of our collection clustered with *Roussoelladoimaesalongensis* with robust support (100% ML, 1 BYPP). *Roussoelladoimaesalongensis* was originally found on decaying bamboo culms in Thailand (Thambugala et al. 2017). Morphologically, our specimens match the description provided by Thambugala et al. (2017) and this species was first reported in China by Seong et al. (2022).

#### Nigrogranaceae Jaklitsch & Voglmayr, 2016

##### ﻿ Nigrograna

Taxon classificationFungiPleosporalesNigrogranaceae

Gruyter, Verkley & Crous, Stud. Mycol. 75: 31 (2012) [2013]

77F81DB5-AC56-543B-9CD5-ADFB23309C88

564794

###### Notes.

*Nigrograna* was described by De Gruyter et al. (2012) as a monotypic genus. *Nigrograna* is characterised by black ascomata, clavate, short pedicellate asci and pale to chocolate brown, asymmetric, fusoid to narrowly ellipsoid, septate ascospores (Zhang et al. 2020a).

##### ﻿ Nigrograna schinifolium

Taxon classificationFungiPleosporalesNigrogranaceae

H. M. Hu & Q. R. Li sp. nov.

316C2F9F-A6F1-556D-B6C1-975C8CF8D254

849204

Fig. 8

###### Type material.

***Holotype*.** GMB0498.

###### Etymology.

With reference to the host, *Zanthoxylumschinifolium* Sieb. & Zucc.

###### Description.

Saprobic on dead stem of *Z. Schinifolium*.

###### Sexual morph:

***Ascomata*** 198–320 μm wide, 105–160 μm high, solitary or aggregated in small groups, black, semi-immersed, appearing as slightly raised regions. ***Ostioles*** are black, lined with paraphyses. ***Peridium*** 26–39 μm wide, comprising several fused layers of "textura angularis", thin-walled and pale brown at the interior, becoming darker and thicker-walled to the outside. ***Hamathecium*** comprising 1–2 μm wide, cylindrical to filiform, septate, branched, pseudoparaphyses, embedded in a gelatinous matrix. ***Asci*** 44–59 × 8–10 μm (average = 51.5 × 9.3 μm, n = 25), 8-spored, bitunicate, fissitunicate, cylindrical to broadly filiform, with a short stipe and knob-like base, apically rounded with a minute ocular chamber. ***Ascospores*** 10–14 × 2.8–4 μm (average = 11.6 × 3.3 μm, n = 40), broadly fusiform to inequilaterally ellipsoid, with the second cell slightly enlarged, straight or slightly curved, with obtuse to rounded ends, hyaline when immature, becoming brown to dark brown at maturity, 3-euseptate, slightly constricted at the median septum. **Asexual morph**: undetermined.

**Figure 8.**
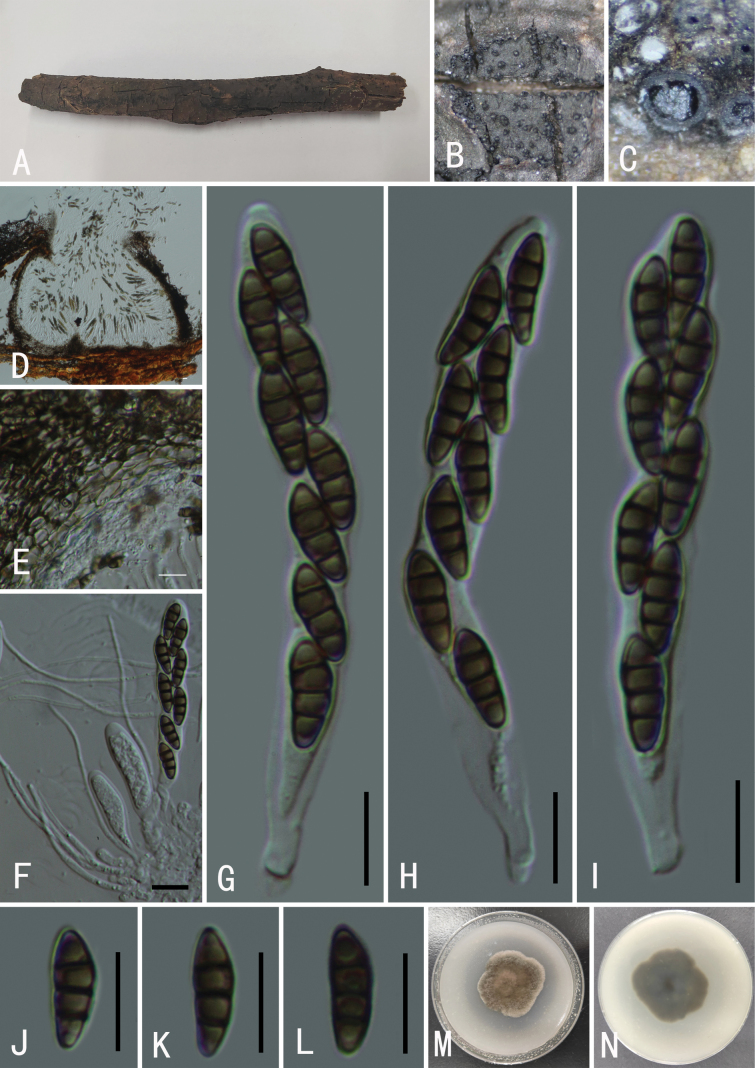
*Nigrogranaschinifolium* (GMB0498) **A** specimen **B** appearance of ascomata on substrate **C** cross-section of ascomata **D** longitudinal section of an ascoma **E** peridium **F** pseudoparaphyses **G**–**I** asci **J–L** ascospores **M, N** culture on PDA. Scale bars: 0.5 mm (**B–C**); 10 μm (**D–L**).

###### Culture characters.

After 4 weeks at 25 °C, colonies on PDA have a diameter of 2–2.5 cm and are circular, slightly raised to umbonate and dull with an entire edge. They appear floccose and smooth and droplets can be observed due to cellular respiration, water formation or antibiotic production. Colonies from the upper region have brown to cream-coloured margins and blackish-brown centres, while their reverse is white to yellowish-brown at the margin and blackish-brown in the centre.

###### Specimen examined.

China, Guizhou Province, Qiannan Prefecture, Sandu Shui Autonomous County, Yao Man Mountain National Forest Park (25°94′18.76"N, 107°95′70.09"E), 563 m elev., on branches of *Zanthoxylumschinifolium*, 28 September 2022, Y.P. Wu, 2022YRS36 (GMB0498, holotype, GMBC0498, ex-type; KUN-HKAS 12983, isotype).

###### Other examined material.

China, Guizhou Province, Huaxi District, Shilihetan Wetland Park (26°23'13.4"N, 106°66'56.4"E), 1501 m elev., on branches of *Zanthoxylumschinifolium*, 8 October 2022, Y.P Wu and H.M Hu, 2022SLHT44 (GMB0504; GMBC0504, living culture).

###### Notes.

*Nigrogranaschinifolium* and *N.thymi* Mapook et al. form a monophyletic clade with moderate support (MPBP 48%, BYPP 0.83, Fig. 3). However, *N.schinifolium* is distinguished by having 3-septate ascospores (Hyde et al. 2017). Morphologically, *N.schinifolium* can be distinguished from other species of *Nigrograna* by its shorter asci and ascospores (Hyde et al. 2017; Zhao et al. 2018; Zhang et al. 2020a). Our research confirms *N.schinifolium* is a new species.

##### ﻿ Nigrograna trachycarpus

Taxon classificationFungiPleosporalesNigrogranaceae

H. M. Hu & Q. R. Li sp. nov.

FF41D3CB-74B3-5ECB-88EB-30AD78E42B1E

849205

Fig. 9

###### Type material.

***Holotype***: GMB0499.

###### Etymology.

Named after the host genus *Trachycarpus* from which the fungus was isolated.

###### Description.

Saprobic or parasitic on dead culms of *Trachycarpus* sp.

**Sexual morph: *Ascomata*** 160–380 μm wide, 100–210 μm high, pyriform to globose, scattered or clustered in small groups, black, immersed, the base remaining immersed in the substrate, smooth, with ostiole. ***Ostiole*** single, central, flattened, with a short neck, without paraphyses. ***Peridium*** 22–34 μm wide, multi-layered, composed of 4–6 rows of heavily pigmented, light brown to dark brown cells of textura angularis. ***Hamathecium*** comprising numerous 1.4–2.2 μm diameter, filamentous, unbranched, anastomosing, septate pseudoparaphyses. ***Asci*** 86–126 × 11–13 μm (average = 99 × 12 μm, n = 25), 8-spored, bitunicate, with fissitunicate dehiscence occurring rarely, elliptical, shortly pedicellate, apically rounded, with an ocular chamber, with a J-subapical ring. ***Ascospores*** 15–17 × 5–7 μm (average = 16.3 × 6.1 μm, n = 40), hyaline to yellow brown, 2–3-septate, deeply constricted at second septum, tapering to each end, the widest point at second cell from apex, smooth-walled, distinctly guttulate, without a sheath or appendages. **Asexual morph**: undetermined.

**Figure 9.**
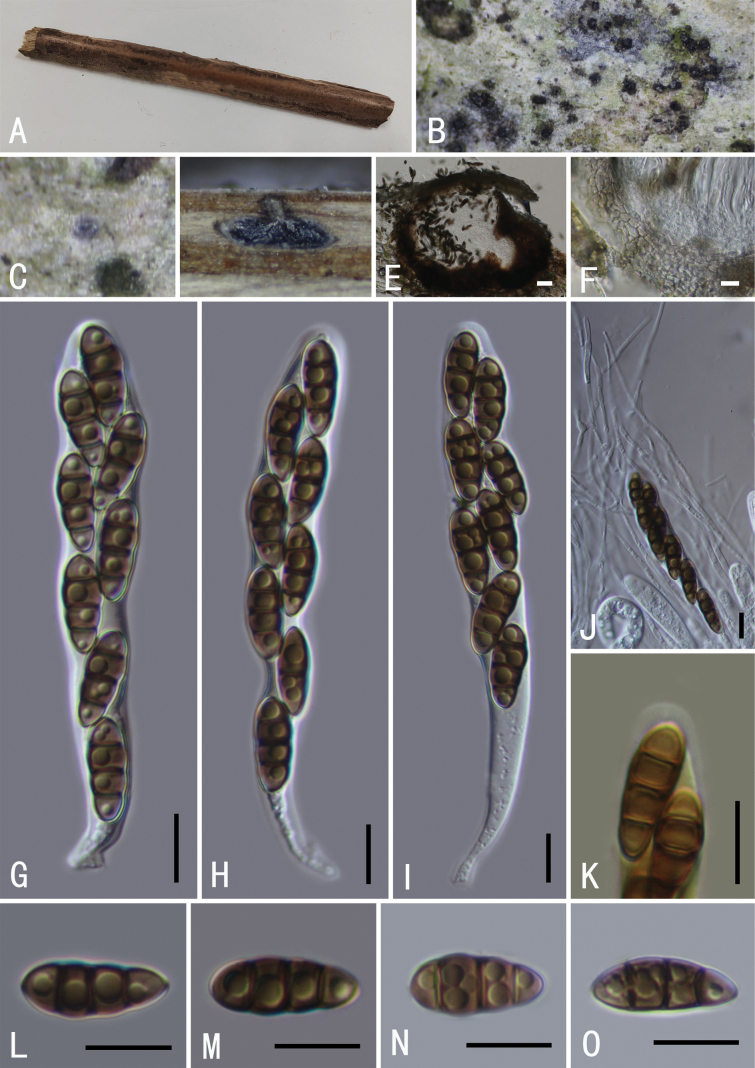
*Nigrogranatrachycarpus* (GMB0499) **A** specimen **B, C** appearance of ascomata on substrate **D, E** longitudinal section of an ascoma **F** peridium **G–I** asci **J** pseudoparaphyses **K** J-ascus subapical ring in Melzer’s **L–O** ascospores. Scale bars: 0.5 mm (**B–D**); 10 μm (**E–O**).

###### Culture characteristics.

After 4 weeks at 25 °C on PDA, colonies typically reach 2–2.5 cm in diameter. They present a circular shape with a dense and elevated centre, while appearing sparse and radiating at the margin. The colonies exhibit colours ranging from dark grey to pale olivaceous when viewed from above and from dark olivaceous to black on reverse.

###### Specimen examined.

China, Guizhou Province, Guiyang Huaxi National Urban Wetland Park (26°2'2.34"N, 106°34'16.22"E), 1130 m elev., on decaying culms of *Trachycarpus* sp., 12 October 2022, Y.P Wu and H.M Hu, 2022 HXGY11 (GMB0499, holotype, GMBC0499, ex-type; KUN-HKAS 12984, isotype).

###### Other examined material.

China, Guizhou Province, Qiannan Prefecture, Sandu Shui Autonomous County, Yao Man Mountain National Forest Park (25°93′18.76"N, 107°95′15.66"E), 540 m elev., on decaying bamboo culms of *Trachycarpus* sp.; 28 September 2022; Y.P. Wu, 2022YRS50 (GMB050; GMBC0505, living culture).

###### Notes.

In the phylogenetic analysis, *Nigrogranatrachycarpus* and *N.locuta-pollinis* F. Liu & L. Cai formed a monophyletic branch within the *Nigrograna* genus, with a bootstrap support value of 31% (Fig. 3). However, this relationship remained consistent in repeated phylogenetic analyses. Sequences generated from the cultures of *N.trachycarpus* are similar to sharing an ITS similarity of 70.7% (with 57/488 gaps) and a tef1 similarity of 89.8% (with 0/481 gaps). Morphologically, *N.trachycarpus* can be distinguished by its larger ascospores, measuring 16.3 × 6.1 μm, in contrast to *N.schinifolium*’s ascospores, 11.6 × 3.3 μm. Morphologically, it is close to *N.impatientis* J.F. Zhang, J.K. Liu & Z.Y. Liu, but the latter typically has ascocarps in groups of 2–6 with ostiole necks penetrating the host surface together. Moreover, the *N.trachycarpus* a possesses longer asci (measuring 99 × 12 μm) and larger ascospores (measuring 16.3 × 6.1 μm) compared to *N.impatientis* (asci measuring 48 × 8, ascospores measuring 12 × 4.3 μm) (Zhang et al. 2020a).

## ﻿Discussion

In this study, based on phylogenetic trees of combined ITS, LSU, SSU, *tef*1 and *rpb*2 sequences and morphology, we described and illustrated three new species of micro-fungi on dead woody litter, viz., *Neokalmusiakarka* (Didymosphaeriaceae), *Nigrogranaschinifolium* and *N.trachycarpus* (Nigrogranaceae) and records of three species of *Roussoella* (Roussoellaceae). Didymosphaeriaceae was introduced by Munk (1953) and is one of the most diverse families within the *Pleosporales*, with a total of 33 genera (Thambugala et al. 2015; Haridas et al. 2020). We included all of these *Didymosphaeriaceae* genera in our phylogenetic analysis. We used a dataset that combines ITS, LSU, SSU, *tef*1 and *rpb*2 genes for this purpose. *Neokalmusia* formed a well-supported monophyletic clade within *Didymosphaeriaceae*, while the newly-discovered species, *N.karka*, exhibited a distinct separation from other known *Neokalmusia* species, supported by strong phylogenetic values.

*Nigrograna*, which is the only genus within *Nigrogranaceae*, is globally distributed and ecologically diverse. Amongst its species, *N.mackinnonii* is the most widely distributed species, mainly found in deciduous forests in Canada and northern USA. *Nigrogranabergmaniae* is mainly distributed in Europe, while *N.novae-zelandiae* was discovered in New Zealand. Approximately one-quarter of existing species live as saprotrophs on the bark or corticated twigs of various hardwoods (Phukhamsakda et al. 2018; Jayasiri et al. 2019). *Nigrogranaschinifolium* was collected from rotten wood, while *N.trachycarpus* was obtained from decaying culms. Notably, several *Nigrograna* species have been established in recent studies without strong bootstrap value support. This finding suggests that these two species, *N.schinifolium* and *N.trachycarpus*, belong to the genus *Nigrograna* with strong evidence supporting this classification.

This study unveils valuable insights about saprophytic fungi, shedding light on their distribution and diversity within the Guizhou Region. It also identified three new species, which are important for the study of fungal taxonomy and further enriches our understanding of these microscopic organisms. Moreover, the study highlights the ongoing instability within the existing taxonomic system, emphasising the necessity for addressing these taxonomic challenges through processes such as re-collection, confirmation and sequencing of samples.

## Supplementary Material

XML Treatment for Neokalmusia

XML Treatment for Neokalmusia karka

XML Treatment for Roussoella

XML Treatment for Roussoella pseudohysterioides

XML Treatment for Roussoella neopustulans

XML Treatment for Roussoella doimaesalongensis

XML Treatment for Nigrograna

XML Treatment for Nigrograna schinifolium

XML Treatment for Nigrograna trachycarpus

## ﻿Additional information

### Author ORCIDs

Hongmin Hu https://orcid.org/0000-0003-3894-3269

Sihan Long https://orcid.org/0000-0002-8346-3646

Nalin N. Wijayawardene https://orcid.org/0000-0003-0522-5498

### Data availability

All of the data that support the findings of this study are available in the main text.
