# ﻿Corrigendum: Hu H et al. (2023) Taxonomic and phylogenetic characterisations of six species of Pleosporales (in Didymosphaeriaceae, Roussoellaceae and Nigrogranaceae) from China. MycoKeys 100: 123–151. https://doi.org/10.3897/mycokeys.100.109423

**DOI:** 10.3897/mycokeys.102.116896

**Published:** 2024-03-07

**Authors:** Hongmin Hu, Minghui He, Youpeng Wu, Sihan Long, Xu Zhang, Lili Liu, Xiangchun Shen, Nalin N. Wijayawardene, Zebin Meng, Qingde Long, Jichuan Kang, Qirui Li

**Affiliations:** 1 State Key Laboratory of Functions and Applications of Medicinal Plants, Guizhou Medical University, Guiyang, Guizhou province, China; 2 The High Efficacy Application of Natural Medicinal Resources Engineering Center of Guizhou Province (The Key Laboratory of Optimal Utilization of Natural Medicine Resources), School of Pharmaceutical Sciences, Guizhou Medical University, University Town, Guian New District, Guiyang, Guizhou province, China; 3 Key Laboratory of Infectious Immune and Antibody Engineering of Guizhou Province, Cellular Immunotherapy Engineering Research Center of Guizhou Province, Immune Cells and Antibody Engineering Research Center of Guizhou Province, School of Biology and Engineering, Guizhou Medical University, Guiyang, Guizhou province, China; 4 Center for Yunnan Plateau Biological Resources Protection and Utilization, College of Biological Resource and Food Engineering, Qujing Normal University, Qujing, Yunnan province, China; 5 Guizhou Tea Seed Resource Utilization Engineering Research Center, Guizhou Education University, Guiyang, Guizhou province, China; 6 The Engineering and Research Center for Southwest Bio-Pharmaceutical Resources of National Education Ministry of China, Guizhou University, Guiyang, Guizhou province, China

Due to an error on our part, we mixed up the figures used in Form 3 of the manuscript, and it was only after the manuscript was published that we noticed that we had misplaced the figures. We therefore provide below a new Table 3 containing the corrected information.

**Table 3.** Taxa and corresponding GenBank accession numbers of sequences used in the phylogenetic analysis of Didymosphaeriaceae, Roussoellaceae and Nigrogranaceae.

Species	Strain	GenBank accession numbers					References
		ITS	SSU	LSU	*tef*1	*rpb*2	
*Alloconiothyriumcamellia*e	NTUCC 17-032-1^T^	MT112294	MT071221	MT071270	MT232967	—	(Kolařík et al. 2017)
*Arthopyrenia* sp.	UTHSC DI16–362	LT796905	LN907505	—	LT797145	LT797065	(Crous et al. 2015)
*Austropleosporaochracea*	KUMCC 20-0020^T^	MT799859	MT808321	MT799860	MT872714	—	(Dissanayake et al. 2021)
*A.keteleeriae*	MFLUCC 18-1551^T^	NR_163349	MK347910	NG_070075	MK360045	—	(Mapook et al. 2020)
*Biatriosporaantibiotica*	CCF 1998	LT221894	—	—	—	—	(Kolařík et al. 2017)
*B.carollii*	CCF 4484^T^	LN626657	—	—	LN626668	—	(Kolařík et al. 2017)
*B.mackinnonii*	E9303e	—	—	—	LN626673	—	(Kolařík et al. 2017)
*B.peruviensis*	CCF 4485^T^	LN626658	—	—	LN626671	—	(Kolařík et al. 2017)
*Bimuriaomanensis*	SQUCC 15280^T^	NR_173301	—	NG_071257	MT279046	—	(Wijesinghe et al. 2020)
*B.novae-zelandiae*	CBS 107.79^T^	MH861181	AY016338	AY016356	DQ471087	—	(Vu et al. 2019)
*Chromolaenicolananensis*	MFLUCC 17-1477	MN325014	MN325008	MN325002	MN335647	—	(Liu et al. 2014)
*C.siamensis*	MFLUCC 17-2527^T^	NR_163337	MK347866	NG_066311	MK360048	—	(Mapook et al. 2020)
*C.thailandensis*	MFLUCC 17-1475	MN325019	MN325013	MN325007	MN335652	—	(Liu et al. 2014)
*C.lampangensis*	MFLUCC 17-1462^T^	MN325016	MN325010	MN325004	MN335649	—	(Liu et al. 2014)
*Cylindroaseptosporaleucaenae*	MFLUCC 17-2424	NR_163333	MK347856	NG_066310	MK360047	—	(Mapook et al. 2020)
*Deniquelatahypolithi*	CBS 146988^T^	MZ064429	—	NG_076735	MZ078250	—	(Ariyawansa et al. 2020b)
*D.barringtoniae*	MFLUCC 16-0271	MH275059	—	MH260291	MH412766	—	(Tibpromma et al. 2018)
*Didymocreasadasivanii*	CBS 438.65	MH858658	DQ384066	DQ384103	—	—	(Vu et al. 2019)
*Didymosphaeriarubi-ulmifolii*	MFLUCC 14-0023^T^	—	NG_063557	KJ436586	—	—	(Jayasiri et al. 2019)
*Kalmusiaerioi*	MFLU 18-0832^T^	MN473058	MN473046	MN473052	MN481599	—	(Vu et al. 2019)
*K.italica*	MFLUCC 13-0066^T^	KP325440	KP325442	KP325441	—	—	(Vu et al. 2019)
*K.variispora*	CBS 121517^T^	NR_145165	NG_070452	—	—	—	(Wijesinghe et al. 2020)
*K.ebuli*	CBS 123120^T^	KF796674	JN851818	JN644073	—	—	(Dissanayake et al. 2021)
*Kalmusibambusatriseptata*	MFLUCC 13-0232	KY682697	KY682696	KY682695	—	—	(Tibpromma et al. 2018)
*Karstenularhodostoma*	CBS 690.94	—	GU296154	GU301821	GU349067	—	(Crous et al. 2021)
*Laburnicolahawksworthii*	MFLUCC 13-0602^T^	KU743194	KU743196	KU743195	—	—	(Ariyawansa et al. 2014)
*Letendraeahelminthicola*	CBS 884.85	MK404145	AY016345	AY016362	MK404174	—	(Tibpromma et al. 2018)
*L.muriformis*	MFLUCC 16-0290^T^	KU743197	KU743199	KU743198	KU743213	—	(Ariyawansa et al. 2014)
*L.padouk*	CBS 485.70	—	GU296162	AY849951	—	—	(Zhang et al. 2013)
*L.cordylinicola*	MFLUCC 11 0148^T^	NR_154118	KM214001	NG_059530	—	—	(Wijayawardene et al. 2020)
*Montagnulachromolaenicola*	MFLUCC 17-1469^T^	NR_168866	NG_070157	NG_070948	MT235773	—	(Liu et al. 2014)
*M.cirsii*	MFLUCC 13 0680	KX274242	KX274255	KX274249	KX284707	—	(Hyde et al. 2020)
*M.krabiensis*	MFLUCC 16-0250^T^	MH275070	MH260343	MH260303	MH412776	—	(Tibpromma et al. 2018)
*M.thailandica*	MFLUCC 17-1508^T^	MT214352	NG_070158	NG_070949	MT235774	—	(Liu et al. 2014)
*M.bellevaliae*	MFLUCC 14-0924^T^	NR_155377	KT443904	KT443902	KX949743	—	(Ariyawansa et al. 2014)
*Neoroussoellaalishanense*	FU31016	MK503816	MK503822	—	MK336181	MN037756	(Verkley et al. 2014)
*N.bambusae*	MFLUCC 11–0124	KJ474827	KJ474839	—	KJ474848	KJ474856	(Dissanayake et al. 2021)
*N.heveae*	MFLUCC 17–1983	MH590693	MH590689	—	—	—	(Wanasinghe et al. 2018)
*N.lenispora*	GZCC 16-0020^T^	—	KX791431	—	—	—	(Hyde et al. 2020)
*N.leucaenae*	MFLUCC 18–1544	MK347767	MK347984	—	MK360067	MK434876	(Mapook et al. 2020)
*N.solani*	CPC 26331^T^	KX228261	KX228312	—	—	—	(Wijayawardene et al. 2014)
*Neokalmusiaarundini*s	MFLUCC 15-0463^T^	NR_165852	NG_068372	NG_068237	KY244024	—	(Thambugala et al. 2015)
*N.brevispora*	KT2313^T^	LC014574	AB524460	AB524601	AB539113	—	(Tanaka et al. 2015)
*N.brevispora*	KT1466	LC014573	AB524459	AB524600	AB539112	—	(Tanaka et al. 2015)
*N.didymospora*	MFLUCC 11-0613	—	KP091435	KP091434	—	—	(Haridas et al. 2020)
*N.jonahhulmei*	KUMCC 21-0819	ON007044	ON007040	ON007049	ON009134	—	(Wanasinghe et al. 2016)
***N.karka***	**GMB0494^T^**	**OR120445**	**OR120442**	**OR120432**	**OR150020**	—	**This study**
***N.karka***	**GMB0500**	**OR120438**	**OR120433**	**OR120443**	**OR150021**	—	**This study**
*N.kunmingensis*	KUMCC 18-0120^T^	MK079886	MK079887	MK079889	MK070172	—	(Vu et al. 2019)
*N.scabrispora*	KT1023	LC014575	AB524452	AB524593	AB539106	—	(Tanaka et al. 2015)
*N.thailandica*	MFLUCC 16-0405^T^	NR_154255	KY706137	NG_059792	KY706145	—	(Thambugala et al. 2015)
*Nigrogranaantibiotica*	CCF 4378^T^	JX570932	—	—	JX570934	—	(Kolařík et al. 2018)
*N.antibiotica*	CCF 1998	LT221894	—	—	—	—	(Kolařík et al. 2018)
*N.cangshanensis*	MFLUCC15-0253^T^	KY511063	—	—	KY511066	—	(Crous et al. 2015)
*N.carollii*	CCF 4484^T^	LN626657	—	—	LN626668	—	(Kolařík et al. 2018)
*N.chromolaenae*	MFLUCC 17-1437^T^	MT214379	—	—	MT235801	—	(Liu et al. 2014)
*N.fuscidula*	CBS 141556^T^	KX650550	—	—	KX650525	—	(Feng et al. 2019)
*N.fuscidula*	CBS 141476	KX650547	—	—	KX650522	—	(Feng et al. 2019)
*N.hydei*	GZCC 19-0050^T^	NR_172415	—	—	MN389249	—	(Zhang et al. 2020)
*N.impatientis*	GZCC 19-0042^T^	NR_172416	—	—	MN389250	—	(Zhang et al. 2020)
*N.locuta-pollinis*	CGMCC 3.18784	MF939601	—	—	MF939613	—	(Ahmed et al. 2014)
*N.locuta-pollinis*	LC11690	MF939603	—	—	MF939614	—	(Ahmed et al. 2014)
*N.mackinnonii*	CBS 674.75^T^	NR_132037	—	—	KF407986	—	(Ariyawansa et al. 2015)
*N.mackinnonii*	E5202H	JX264157	—	—	JX264154	—	(Phukhamsakda et al. 2018)
*N.mackinnonii*	E9303e	—	—	—	LN626673	—	(Kolařík et al. 2017)
*N.magnoliae*	GZCC 17-0057	MF399066	—	—	MF498583	—	(Zhang et al. 2020)
*N.magnoliae*	MFLUCC 20-0020^T^	MT159628	—	—	MT159605	—	(Liu et al. 2014)
*N.mycophila*	CBS 141478^T^	KX650553	—	—	KX650526	—	(Feng et al. 2019)
*N.mycophila*	CBS 141483	KX650555	—	—	KX650528	—	(Feng et al. 2019)
*N.norvegica*	CBS 141485^T^	KX650556	—	—	—	—	(Feng et al. 2019)
*N.obliqua*	CBS 141477^T^	KX650560	—	—	KX650531	—	(Feng et al. 2019)
*N.obliqua*	CBS 141475	KX650558	—	—	KX650530	—	(Feng et al. 2019)
*N.peruviensis*	CCF 4485^T^	LN626658	—	—	LN626671	—	(Kolařík et al. 2018)
*N.rhizophorae*	MFLUCC 18-0397^T^	MN047085	—	—	MN077064	—	(Poli et al. 2020)
*N.samueliana*	NFCCI-4383^T^	MK358817	—	—	MK330937	—	(Poli et al. 2020)
***N.schinifolium***	**GMB0498^T^**	**OR120434**	—	—	**OR150022**	—	**This study**
***N.schinifolium***	**GMB0504**	**OR120441**	—	—	**OR150023**	—	**This study**
*N.thymi*	MFLUCC 14-1096^T^	KY775576	—	—	KY775578	—	(Crous et al. 2015)
***N.trachycarpus***	**GMB0499^T^**	**OR120437**	—	—	**OR150024**	—	**This study**
***N.trachycarpus***	**GMB0505**	**OR120440**	—	—	**OR150025**	—	**This study**
*N.yasuniana*	YU.101026^T^	HQ108005	—	—	LN626670	—	(Kolařík et al. 2018)
*Occultibambusapustula*	MFLUCC 11-0502^T^	KU940126	—	—	—	—	(Crous et al. 2014)
*O.bambusae*	MFLUCC 13-0855^T^	KU940123	—	—	KU940193	—	(Crous et al. 2014)
*Paracamarosporiumfagi*	CPC 24890^T^	NR_154318	—	NG_070630	—	—	(Ariyawansa et al. 2014)
*P.cyclothyrioides*	CBS 972.95	JX496119	AY642524	JX496232	—	—	(Schoch et al. 2009)
*P.estuarinum*	CBS 109850^T^	JX496016	AY642522	JX496129	—	—	(Verkley et al. 2014)
*P.hawaiiense*	CBS 120025^T^	JX496027	EU295655	JX496140	—	—	(Verkley et al. 2014)
*P.robiniae*	MFLUCC 14–1119^T^	KY511142	KY511141	—	KY549682	—	(Crous et al. 2015)
*P.rosarum*	MFLUCC 17–6054^T^	NR_157529	NG_059872	—	MG829224	—	(Hyde et al. 2016)
*P.rosicola*	MFLUCC 15-0042	NR_157528	MG829153	MG829047	—	—	(Hyde et al. 2016)
*Paramassariosphaeriaanthostomoides*	CBS 615.86	MH862005	GU205246	GU205223	—	—	(Vu et al. 2019)
*Paraphaeosphaeriarosae*	MFLUCC 17-2547^T^	MG828935	MG829150	MG829044	MG829222	—	(Hyde et al. 2016)
*Pararoussoellamukdahanensis*	KUMCC 18-0121	MH453489	MH453485	—	MH453478	MH453482	(Flakus et al. 2019)
*Parathyridariaramulicola*	CBS 141479^T^	KX650565	KX650565	—	KX650536	KX650584	(Feng et al. 2019)
*Phaeodothiswinteri*	CBS 182.58	—	GU296183	GU301857	—	—	(Zhang et al. 2013)
*Pseudocamarosporiumpropinquum*	MFLUCC 13-0544^T^	KJ747049	KJ819949	KJ813280	—	—	(Thambugala et al. 2017)
*Pseudodidymocyrtislobariellae*	KRAM Flakus 25130^T^	NR_169714	NG_070349	NG_068933	—	—	(Tanaka et al. 2015)
*Pseudoneoconiothyriumeuonymi*	CBS 143426^T^	MH107915	MH107961	—	—	MH108007	(Valenzuela-Lopez et al. 2017)
*Pseudopithomycesentadae*	MFLUCC 17-0917^T^	—	MK347835	NG_066305	MK360083	—	(Mapook et al. 2020)
*Pseudoroussoellachromolaenae*	MFLUCC 17–1492^T^	MT214345	MT214439	—	MT235769	—	(Liu et al. 2014)
*P.elaeicola*	MFLUCC 15–0276a	MH742329	MH742326	—	—	—	(Liu et al. 2014)
*P.kunmingnensis*	MFLUCC 17-0314	MF173607	MF173606	MF173605	—	—	(Mapook et al. 2020)
*P.pteleae*	MFLUCC 17-0724^T^	NR_157536	MG829166	MG829061	MG829233	—	(Hyde et al. 2016)
*P.rosae*	MFLUCC 15-0035^T^	MG828953	MG829168	MG829064	—	—	(Hyde et al. 2016)
*P.ulmi-minoris*	MFLUCC 17-0671^T^	NR_157537	MG829167	MG829062	—	—	(Hyde et al. 2016)
*Roussoellaacaciae*	CBS:138873^T^	KP004469	KP004497	—	—	—	(Karunarathna et al. 2019)
*R.aquatic*	MFLUCC 18-1040^T^	NR171975	NG073797	—	—	—	(Liu et al. 2014)
*R.chiangraina*	MFLUCC 10-0556^T^	NR155712	NG059510	—	—	—	(Dissanayake et al. 2021)
*R.doimaesalongensis*	MFLUCC 14-0584^T^	NR165856	NG068241	—	KY651249	KY678394	(Thambugala et al. 2015)
***R.doimaesalongensis***	**GMB0497**	**OR116188**	**OR117732**	—	**OR150026**	—	**This study**
***R.doimaesalongensis***	**GMB0503**	**OR120435**	**OR120444**	—	**OR150027**	—	**This study**
*R.elaeicola*	MFLUCC 15-15-0276a	MH742329	MH742326	—	—	—	(Crous et al. 2015)
*R.euonymi*	CBS:143426^T^	MH107915	MH107961	—	—	MH108007	(Valenzuela-Lopez et al. 2017)
*R.guttulata*	MFLUCC 20-0102^T^	NR172428	NG075383	—	—		(Senwanna et al. 2018)
*R.hysterioides*	CBS 546.94	MH862484	MH874129	—	KF443399	KF443392	(Vilgalys et al. 1990)
*R.intermedia*	CBS 170.96	KF443407	KF443382	—	KF443398	KF443394	(Crous et al. 2013)
*R.japanensis*	MAFF 239636^T^	NR155713	—	—	—	—	(Dissanayake et al. 2021)
*R.kunmingensis*	HKAS 101773^T^	MH453491	MH453487	—	MH453480	MH453484	(Flakus et al. 2019)
*R.magnatum*	MFLUCC 15-0185^T^	—	KT281980	—	—	—	(Jiang et al. 2019)
*R.mangrovei*	MFLU 17-1542^T^	MH025951	MH023318	—	MH028246	MH028250	(Jaklitsch and Voglmayr 2016)
*R.margidorensis*	MUT 5329^T^	NR169906	MN556322	—	MN605897	MN605917	(Tibpromma et al. 2017)
*R.mediterranea*	MUT5369^T^	KU314947	MN556324	—	MN605899	MN605919	(Tibpromma et al. 2017)
*R.mexicana*	CPC 25355^T^	KT950848	KT950862	—	—	—	(Crous et al. 2015a)
*R.mukdahanensis*	MFLU 11-0237^T^	NR155722	—	—	—	—	(Crous et al. 2014)
*R.multiplex*	GMB0316^T^	ON479891	—	ON479892	—	—	(Dong et al. 2020)
*R.neopustulans*	MFLUCC 11-0609^T^	KJ474833	KJ474841	—	KJ474850	—	(Dissanayake et al. 2021)
***R.neopustulans***	**GMB0496**	**OR120436**	**OR120446**				**This study**
***R.neopustulans***	**GMB0502**	**OR116176**	**OR117714**				**This study**
*R.nitidula*	MFLUCC 11-0634	KJ474834	KJ474842	—	KJ474851	KJ474858	(Dissanayake et al. 2021)
*R.padinae*	MUT 5503^T^	—	MN556327	—	MN605902	MN605922	(Tibpromma et al. 2017)
*R.percutanea*	CBS 868.95	KF322118	KF366449	—	KF407987	KF366452	(Ahmed et al. 2014a)
*R.pseudohysterioides*	GMBC0009^T^	MW881445	MW881451	—	—	MW883345	(Zhang et al. 2020)
***R.pseudohysterioides***	**GMB0495**	**OR116175**	**OR117737**	—	**OR150028**	—	**This study**
***R.pseudohysterioides***	**GMB0501**	**OR120447**	**OR120439**	—	**OR150029**	—	**This study**
*R.pustulans*	KT 1709	—	AB524623	—	AB539116	AB539103	(Zhang et al. 2020)
*R.scabrispora*	MFLUCC 14-0582	KY026583	KY000660	—	—	—	(Zhang et al. 2020)
*R.siamensis*	MFLUCC 11-0149^T^	KJ474837	KJ474845	—	KJ474854	KJ474861	(Dissanayake et al. 2021)
*R.thailandica*	MFLUCC 11-0621^T^	KJ474838	KJ474846	—	—	—	(Dissanayake et al. 2021)
*R.tuberculata*	MFLUCC 13-0854^T^	KU940132	KU863121	—	KU940199		(Crous et al. 2014)
*R.verrucispora*	CBS 125434^T^	KJ474832	—	—	—	—	(Dissanayake et al. 2021)
*R.yunnanensis*	HKAS 101762	MH453492	MH453488	—	MH453481	—	(Flakus et al. 2019)
*Roussoellopsismacrospora*	MFLUCC 12-0005	—	KJ474847	—	KJ474855	KJ474862	(Dissanayake et al. 2021)
*R.tosaensis*	KT 1659	—	AB524625	—	AB539117	AB539104	(Zhang et al. 2020)
*Setoarthopyreniachromolaenae*	MFLUCC 17–1444	MT214344	MT214438	—	MT235768	MT235805	(Liu et al. 2014)
*Spegazziniadeightonii*	yone 212	—	AB797292	AB807582	AB808558	—	(Tanaka et al. 2015)
*S.radermacherae*	MFLUCC 17-2285^T^	MK347740	MK347848	MK347957	MK360088	—	(Mapook et al. 2020)
*S.tessarthra*	NRRL 54913	JQ673429	AB797294	AB807584	AB808560	—	(Tanaka et al. 2015)
*Thyridariaacaciae*	CBS 138873	KP004469	KP004497	—	—	—	(Liu et al. 2014)
*T.broussonetiae*	CBS 141481	NR_147658	KX650568	—	KX650539	KX650586	(Karunarathna et al. 2019)
*Torulaherbarum*	CBS 111855	KF443409	KF443386	—	KF443403	KF443396	(Crous et al. 2013)
*T.hollandica*	CBS 220.69	KF443406	KF443384	—	—	KF443393	(Crous et al. 2013)
*Tremateiaarundicola*	MFLU 16-1275	KX274241	KX274254	KX274248	KX284706	—	(Hyde et al. 2020)
*T.chromolaenae*	MFLUCC 17-1425^T^	NR_168868	NG_070160	NG_068710	MT235778	—	(Tanaka et al. 2015)
*T.guiyangensis*	GZAAS01	KX274240	KX274253	KX274247	KX284705	—	(Hyde et al. 2020)
*T.murispora*	GZCC 18-2787	NR_165916	MK972750	MK972751	MK986482	—	(Feng et al. 2019)
*T.thailandensis*	MFLUCC 17-1430^T^	NR_168869	NG_070161	NG_068711	MT235781	—	(Liu et al. 2014)
*Verrucoconiothyriumnitidae*	CBS:119209	EU552112	—	EU552112	—	—	(Wanasinghe et al. 2018)
*Xenocamarosporiumacaciae*	CPC 24755^T^	NR_137982	—	NG_058163	—	—	(Crous et al. 2015b)
*Xenoroussoellatriseptata*	MFLUCC 17–1438	MT214343	MT214437	—	MT235767	MT235804	(Liu et al. 2014)

## ﻿Additional information

### Author ORCIDs

Hongmin Hu https://orcid.org/0000-0003-3894-3269

Sihan Long https://orcid.org/0000-0002-8346-3646

Nalin N. Wijayawardene https://orcid.org/0000-0003-0522-5498

Jichuan Kang https://orcid.org/0000-0002-6294-5793

Qirui Li https://orcid.org/0000-0001-8735-2890

### Data availability

All of the data that support the findings of this study are available in the main text.

